# Longitudinal changes in cocaine intake and cognition are linked to cortical thickness adaptations in cocaine users

**DOI:** 10.1016/j.nicl.2019.101652

**Published:** 2019-01-04

**Authors:** Sarah Hirsiger, Jürgen Hänggi, Jürgen Germann, Matthias Vonmoos, Katrin H. Preller, Etna J.E. Engeli, Matthias Kirschner, Caroline Reinhard, Lea M. Hulka, Markus R. Baumgartner, Mallar M. Chakravarty, Erich Seifritz, Marcus Herdener, Boris B. Quednow

**Affiliations:** a Experimental and Clinical Pharmacopsychology, Department of Psychiatry, Psychotherapy, and Psychosomatics, Psychiatric Hospital, University of Zurich, Zurich, Switzerland; b Division Neuropsychology, Department of Psychology, University of Zurich, Zurich, Switzerland; c Cerebral Imaging Center, Douglas Mental Health University Institute, McGill University, Montreal, QC, Canada; d Center for Addictive Disorders, Department of Psychiatry, Psychotherapy, and Psychosomatics, Psychiatric Hospital, University of Zurich, Zurich, Switzerland; e Zurich Center for Integrative Human Physiology, University of Zurich, Zurich, Switzerland; f Center of Forensic Hairanalytics, Institute of Forensic Medicine, University of Zurich, Zurich, Switzerland; g Departments of Psychiatry and Biomedical and Biological Engineering, McGill University, Montreal, QC, Canada; h Department of Psychiatry, Psychotherapy, and Psychosomatics, Psychiatric Hospital, University of Zurich, Zurich, Switzerland; i Neuroscience Center Zurich, University of Zurich and Swiss Federal Institute of Technology Zurich, Zurich, Switzerland

**Keywords:** Cognition, Endophenotype, Cocaine-related disorders, Prefrontal cortex

## Abstract

**Background:**

Cocaine use has been consistently associated with decreased gray matter volumes in the prefrontal cortex. However, it is unclear if such neuroanatomical abnormalities depict either pre-existing vulnerability markers or drug-induced consequences. Thus, this longitudinal MRI study investigated neuroplasticity and cognitive changes in relation to altered cocaine intake.

**Methods:**

Surface-based morphometry, cocaine hair concentration, and cognitive performance were measured in 29 cocaine users (CU) and 38 matched controls at baseline and follow-up. Based on changes in hair cocaine concentration, CU were classified either as *Decreasers* (*n* = 15) or *Sustained Users* (*n* = 14). Surface-based morphometry measures did not include regional tissue volumes.

**Results:**

At baseline, CU displayed reduced cortical thickness (CT) in lateral frontal regions, and smaller cortical surface area (CSA) in the anterior cingulate cortex, compared to controls. In *Decreasers*, CT of the lateral frontal cortex increased whereas CT within the same regions tended to further decrease in *Sustained Users*. In contrast, no changes were found for CSA and subcortical structures. Changes in CT were linked to cognitive performance changes and amount of cocaine consumed over the study period.

**Conclusions:**

These results suggest that frontal abnormalities in CU are partially drug-induced and can recover with decreased substance use. Moreover, recovery of frontal CT is accompanied by improved cognitive performance confirming that cognitive decline associated with cocaine use is potentially reversible.

## Highlights

• Thickness of the lateral frontal cortex was reduced in cocaine users at baseline.
• Frontal lobe cortical thickness recovered after decreased cocaine use at follow-up.
• Recovery in gray matter structures was related to improved cognitive performance.
• No change was found for cortical surface area and subcortical structures.

## 1 Introduction

Results from recent wastewater analyses from 2017 have revealed a worrying picture about cocaine consumption in Europe as they emphasize a strong upward trend for detected benzoylecgonine (main metabolite of cocaine) levels across many European cities (EMCDDA, 2018). These numbers are alarming, as cocaine consumption is associated with several long-term health conditions along with disrupted social relationships, thus, leading to a high burden for society and economy (Degenhardt and Hall, 2012; Nutt et al., 2007). Beyond that, cocaine use is linked to considerable cognitive deficits (Spronk et al., 2013; Vonmoos et al., 2013) and alterations in brain structures (Ersche et al., 2013b; Mackey and Paulus, 2013). Diminished gray matter (GM) volumes have been recurrently reported especially in frontal brain areas, insula, and the thalamus (e.g., Ersche et al., 2011; Kaag et al., 2014; Lim et al., 2008; Sim et al., 2007). Specifically, GM alterations in frontal regions have been associated with cognitive functioning (Hanlon et al., 2011), decision-making performance (Tanabe et al., 2009), and trait impulsivity of chronic cocaine users (CU) (Crunelle et al., 2014; Moreno-Lopez et al., 2012).

Although there is consensus about reduced cortical GM volumes in chronic CU, results for subcortical volumes, especially for the striatum, are inconsistent and conflicting. Comparing data between dependent CU and healthy controls, spatially distinct increases in striatal GM volume (Ersche et al., 2011), smaller GM volumes in the striatum (Barros-Loscertales et al., 2011), and also the absence of any differences have been reported (Garza-Villarreal et al., 2016). Cross-sectional neuroanatomical alterations seen in CU are commonly interpreted as a consequence of cocaine use as studies found associations between the duration of cocaine intake and GM abnormalities (Barros-Loscertales et al., 2011; Ersche et al., 2011; Ide et al., 2014). This claim is partly supported by a recent longitudinal study demonstrating that reduced or ceased cocaine intake over a study period of six month was associated with an increase in GM volume in the inferior frontal gyrus (IFG) and ventromedial prefrontal regions (Parvaz et al., 2017). Additionally, increased GM volume of the IFG was positively associated with cognitive flexibility performance, which was examined only in CU. Furthermore, cocaine use intensity of the participants was only assessed by self-reports. Recovery of cognitive performance after ceasing cocaine intake was previously shown by our laboratory especially for attention and memory performance (Vonmoos et al., 2014). The same longitudinal study also found evidence that increased cocaine intake was significantly associated with reduced cognitive performance especially for working memory (Vonmoos et al., 2014).

However, there is a second possible interpretation of the reported cross-sectional GM alterations. Next to substance-induced plasticity, these differences in brain structures might depict pre-existing vulnerability markers, a possibility discussed in a recent sibling study. While both siblings, one addicted to cocaine whereas the other had no history of drug intake, showed increased GM volume in subcortical regions compared to non-related controls, a decrease in GM volume in the prefrontal cortex was only applicable in the cocaine dependent sibling (Ersche et al., 2012). As brain structures are suggested to be highly heritable (Wright et al., 2002), the authors concluded that the subcortical enlargements are potential endophenotypes for cocaine addiction.

Taken together, it is not fully understood whether cortical alterations found in CU are predisposed or cocaine-induced (or both). Moreover, it is unclear how prolonged use of cocaine, in contrast to a reduction in cocaine intake affects GM. Finally, the neuronal basis of well-documented cognitive deficits in CU is not yet clear (Vonmoos et al., 2014). In this current longitudinal study we therefore collected data from stimulant-naïve controls and CU with reduced/ceased but also with sustained/increased cocaine use at baseline and after a time interval of at least six and up to 53 months. The group categorization was built on objective toxicological hair analyses covering the use of the last 6 months. Surface-based morphometry measures of six regions of interest in the frontal lobe and volumetric measures of subcortical structures were analyzed. In contrast to voxel-based morphometry, surface-based morphometry offers the possibility to analyze cortical thickness (CT) and cortical surface area (CSA) separately. Disentangling these two parameters is important, as CT and CSA have been described as having minimal genetic relationship and stand for different facets of GM (Rakic, 1988, Rakic, 1995, Rakic, 2007). Thus, we anticipated finding new information about possible neuroplasticity not only in participants decreasing but also in participants with sustained cocaine consumption. With additional data on key cognitive functions (sustained attention and working memory), we aimed to gain more insight into the possible neuroanatomical underpinnings of cognitive deficits in CU.

We hypothesized CT/CSA in the six frontal regions of interest and cognitive performance to be lower in CU than in controls at baseline. Moreover, severity of drug use was expected to correlate negatively with cortical variables. In contrast, positive associations were anticipated between cognitive performance and cortical parameters. We further hypothesized dose-dependent negative alterations in frontal regions and in accordance with Parvaz et al. (2017) we expected that CT and CSA are negatively affected by cocaine consumption and that those changes were more pronounced with higher amount of cocaine intake during the study interval. Moreover, cortical changes were also expected to be positively associated with cognitive change scores. Subcortical structures were analyzed in an explorative fashion as no clear picture could be drawn from the previous literature.

## 2 Material and methods

### 2.1 Participants

A total of 38 healthy controls and 29 CU were included in this study (for recruitment and selection details see Suppl. Methods 1). To be eligible to participate CU had to report cocaine as primary drug of use with a consumption level of >0.5 g per month, and an abstinence duration of <6 months to ensure regular and recent use to ensure that all the participants were current users at baseline. Exclusion criteria for CU were the presence of DSM-IV Axis I adult psychiatric disorders—except for cocaine, cannabis, nicotine, alcohol abuse/dependence, attention deficit hyperactivity disorder (ADHD), and a previous depressive episode. Controls were excluded if they displayed a current or previous DSM-IV Axis I psychiatric disorders (except for nicotine dependence), and illegal drug use of >15 lifetime occasions or during the past 6 months with the exception of cannabis. Before the testing session, participants were asked to abstain from illegal substances for at least 72 h and not to consume alcohol for 24 h. Urine samples were collected to verify self-reports. When available, 6 cm hair samples were cut from the occiput enabling to objectively estimate drug use during the last six months. Hair samples were analyzed with liquid chromatography-tandem mass spectrometry (see Suppl. Methods 2). Data from these 6-months hair samples were used for a) confirmation of regular cocaine use (participants were only included if cocaine hair concentration was at least 0.5 ng/mg, an excepted threshold for reliable cocaine detection; Bush, 2008; Cooper et al., 2012); b) confirmation that cocaine is the primary used illicit drug, and c) for separation of the CU into two groups (see Suppl. Methods 2 for urine and hair toxicology analyses). Participants were mostly right handed (94.0%) and there was no group difference in handedness (x^2^_2_ = 2.49, P = .29). The ethics committee of the Canton of Zurich approved the study, which was in accordance with guidelines from the Helsinki declaration. All participants gave written informed consent and were compensated for their participation.

### 2.2 Group assignment

The included 29 CU were classified into two groups using hair concentration for cocaine and related metabolites: *Decreasers* or *Sustained Users* (Suppl. Fig. 1). Participants were categorized as *Decreasers* based on a combination of an absolute and relative change in their hair cocaine parameters from baseline to follow-up: There had to be an absolute decrease in cocaine of at least 0.5 ng/mg from baseline to follow-up and the more robust parameter cocaine_total_ (=cocaine + benzoylecgonine + norcocaine; Hoelzle et al., 2008) had to be at least 10% less at follow-up than at baseline (criteria based on Vonmoos et al., 2014). The *Sustained Users* included participants, which did either not change their consumption behavior or increased their cocaine intake based on cocaine_total_ levels. In addition, the *Sustained Users* also included one participant meeting the criterion for the *Decreasers* but had a measured cocaine_total_ concentration at the follow-up time point which was above a value of 27.8 ng/mg. This threshold was chosen in accordance to a study, which showed that dependent CU had on average hair cocaine_total_ concentrations of 27.8 ng/mg (Vonmoos et al., 2013). With a sustained dependent consumption behavior we did not expect an improvement either in structural brain measures or cognitive performance. For three CU (1 *Decreaser*, 2 *Sustained Users*) hair samples were not available at baseline. Those participants were either categorized with the help of the amount of cocaine_total_ concentration measured at follow-up (two had larger concentration of cocaine_total_ than 27.8 ng/mg, thus were categorized as *Sustained Users*) and/or the reported cocaine use at baseline versus follow-up (one participant with cocaine_total_ of 2.15 ng/mg at follow-up reported decreased cocaine consumption at the second time point, thus was categorized as *Decreaser*). For one participant, no hair sample could be collected at any time point. Due to his reported consistent high cocaine consumption of >30 g per week, the participant was classified as *Sustained User*. Based on these stipulations 15 CU were categorized as *Decreasers* and 14 were classified as *Sustained Users*.

**Fig. 1:**
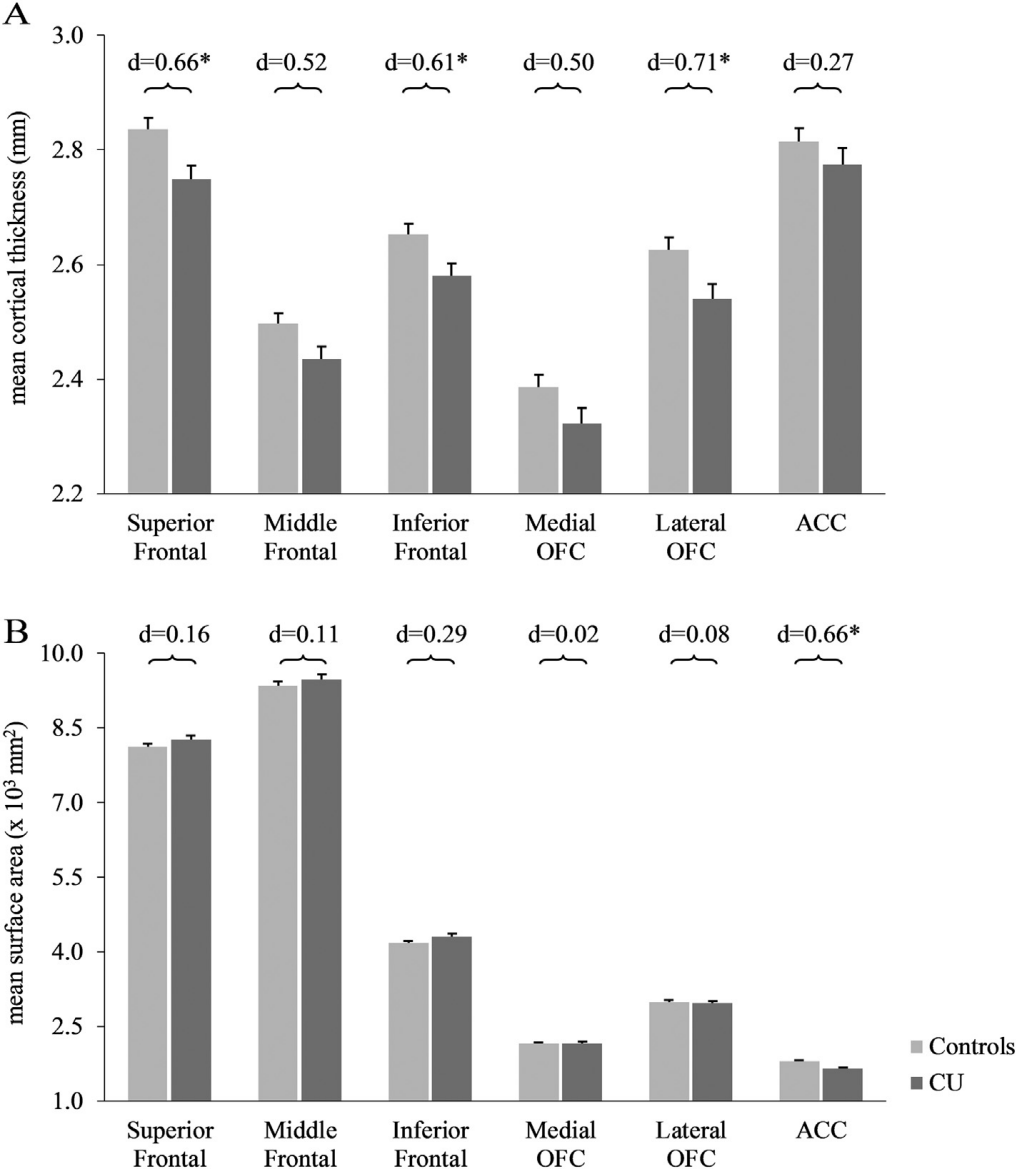
Cortical group differences at baseline. Group differences for cortical measures for the six regions of interest. (A) Mean cortical thickness (in mm) and standard errors. All values corrected for age, verbal IQ, and ADHD-SR score. (B) Mean cortical surface area (×10^3^ in mm^2^) and standard errors. All values corrected for age, verbal IQ, ADHD-SR score, and total surface area. *P < .05 corrected for multiple comparisons. Cohen's d controls vs. cocaine users.

### 2.3 Procedure

After the baseline measurement a subsequent follow-up data collection was conducted after a minimum of 6 months (interval duration: M = 19 months, max. duration 53 months). At both time points, MRI scans as well as demographical information and drug use parameters were collected at the same day. A trained psychologist conducted a clinical interview to determine the presence of DSM-IV Axis I diagnoses (MINI/SCID-I; APA, 1994). Beyond hair testing, all subjective data on drug use was assessed by the Interview for Psychotropic Drug Consumption (Quednow et al., 2004). In addition, the brief version of the Cocaine Craving Questionnaire (CCQ) was used to identify current cocaine craving (Sussner et al., 2006). Participants were additionally asked to complete the Beck Depression Inventory (BDI; Beck et al., 1961) to reveal current severity of depression. Smoking habits were assessed by the use of the Fagerström Test of Nicotine Dependence (Heatherton et al., 1991). Lastly, a standard German vocabulary test to estimate verbal intelligence (IQ; Lehrl, 1999) and the ADHD self-rating scale (ADHD-SR; Roesler et al., 2004) to estimate ADHS symptomatology, were applied. As we did not expect changes in ADHD ratings and verbal intellectual performance over the study period, both questionnaires were only carried out at baseline.

Cognitive data for sustained attention (*n* = 51) and working memory (*n* = 63) performance was available for a large subset of the included participants. Attention and working memory performance were investigated because those “top-down” cognitive abilities are strongly linked to frontal cortex functioning (D'Esposito and Postle, 1999; Pardo et al., 1991). Moreover, previous longitudinal data from our laboratory revealed that increased cocaine use was particularly associated with increased deficits in working memory performance (Vonmoos et al., 2014). Recreational CU displayed the strongest impairments in cognitive tasks measuring sustained attention and in turn fully recovered from attention deficits when quitting their cocaine intake completely at follow-up (Vonmoos et al., 2013, Vonmoos et al., 2014). Sustained attention performance was assessed by the discrimination performance A` measured with the Rapid Visual Processing (RVP) test from the Cambridge Neuropsychological Test Automated Battery (CANTAB, http://www.cantab.com). For the RVP task, 28 controls, 12 *Decreasers*, and 11 *Sustained Users* had available data at both measurement points. Verbal working memory was measured by the sum score of the Letter Number Sequencing Task (LNST, Wechsler, 1997) and was completed by a subset of 63 participants (37 controls, 14 *Decreasers*, and 12 *Sustained Users*). At baseline cognitive tests were conducted on average 17 weeks (RVP) and 14 weeks (LNST) prior to MRI acquisition. There were no group differences in time between cognitive testing and MRI acquisition for both cognitive tasks (RVP: F_2,48_ = 1.21, P = .31, _p_η^2^ = 0.05; LNST: F_2,60_ = 1.68, P = .20, _p_η^2^ = 0.05). At follow-up, cognitive and MRI data were collected at the same day (additional information about the procedure can be found in Suppl. Methods 3).

### 2.4 Image data acquisition

At both time points, MRI data for all subjects were collected on the same 3 T Philips Achieva whole-body scanner equipped with a 32-channel receive head coil, using the same standard T1-weighted 3D magnetization-prepared rapid gradient echo (MPRAGE) pulse sequence. Image parameter were: repetition time (TR) = 8.08 ms, echo time (TE) = 3.7 ms, field of view (FOV) = 240 × 240 mm^2^, 160 slices, voxel size of 1 × 1 × 1 mm^3^.

### 2.5 Surface-based MRI data processing

To evaluate changes in CT and CSA FreeSurfer (v5.3.0) was used. FreeSurfer is a freely available (http://surfer.nmr.mgh.harvard.edu/), automated, image analysis suit that has been well documented in former publications (Dale et al., 1999; Fischl and Dale, 2000; Fischl et al., 1999, Fischl et al., 2004). For more details on MRI data processing please see Suppl. Methods 4. After cross-sectional preprocessing, FreeSurfer's already established longitudinal pipeline (Reuter et al., 2010, Reuter et al., 2012), previously shown to be highly reliable (Liem et al., 2014), was applied. To extract CT and CSA for several regions of interests (ROI), the cortex was parcellated by the use of the Desikan-Killiany Atlas (Desikan et al., 2006). As most pronounced alterations between CU and healthy controls were reported within the frontal lobe, we restricted our analysis to ROI within this area (Ersche et al., 2013b). Thus, our analysis included the superior frontal gyrus (SFG), middle frontal gyrus (MFG; caudal and rostral middle frontal gyrus), IFG (pars opercularis, orbitalis, and triangularis), medial orbitofrontal cortex (OFC), lateral OFC, and anterior cingulate cortex (ACC; caudal and rostral anterior cingulate cortex). Either the mean (for CT) or the sum (for CSA) of the mentioned subparts for each ROI was calculated. As ROI in both hemispheres were significantly correlated (see Suppl. Methods 5) we averaged the CT and CSA for each ROI from both hemispheres to reduce the number of comparisons. Moreover, as we aimed to analyze CT and CSA separately we refrained to weight the ROIs for the CSA when analyzing CT, as we were afraid to blur information from CSA into our CT analyses (for more information see Suppl. Methods 4). See Suppl. Fig. 2 for the six cortical ROI used in this study.

**Fig. 2:**
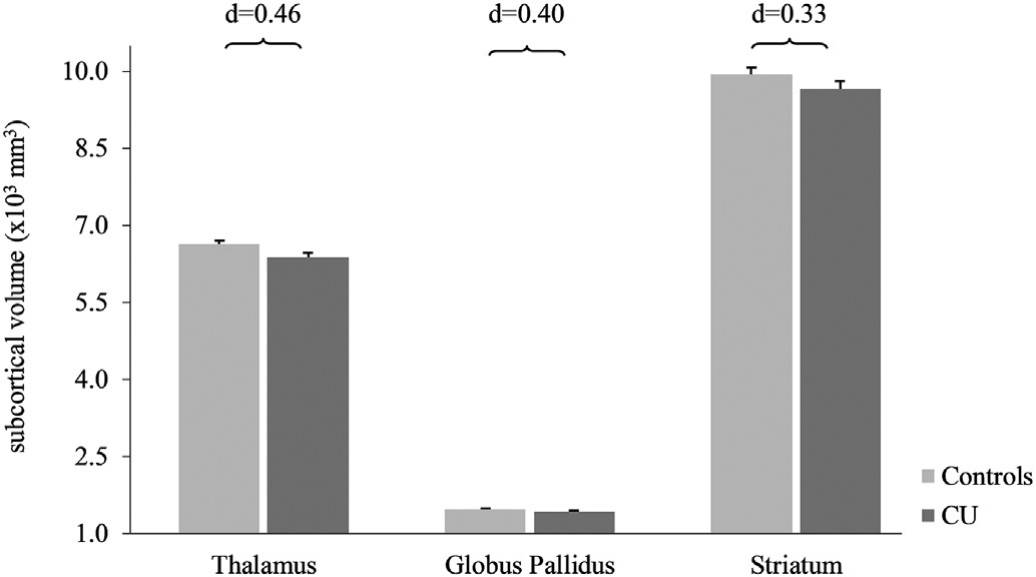
Subcortical measures at baseline. Subcortical mean volume measures (×10^3^ in mm^3^) and standard errors in cocaine users and controls. All values corrected for age, verbal IQ, ADHD-SR score, and ICV. None of the group differences survived multiple comparisons correction. Cohen's d controls vs. cocaine users.

### 2.6 Subcortical volumetric MRI data processing

To analyze subcortical structures the MAGeTbrain-Toolbox (Chakravarty et al., 2013) was used. This automatic segmentation algorithm was employed because it has recently been shown to be nearly as accurate as segmentations done by elaborate and costly manual tracing (the “gold standard” of segmentation; Chakravarty et al., 2013; Makowski et al., 2018). In addition, it has been reported to be more precise than other tools such as FreeSurfer and FSL FIRST (Makowski et al., 2018). All T1-weighted images were first preprocessed (non-uniformity correction (iterative n4), brainextraction, cropping) using the minc-bpipe-library (https://github.com/CobraLab/minc-bpipe-library). The preprocessed images were then further segmented using the MAGeTbrain-Toolbox (Chakravarty et al., 2013) to extract volume measures for the thalamus, globus pallidus, and striatum (https://github.com/CobraLab/MAGeTbrain). For more information regarding data processing please see Suppl. Methods 4. As for the surface-based neuroanatomical parameters, volumes for subcortical structures were averaged for the left and right hemisphere (correlations are reported in the Suppl. Methods 5).

### 2.7 Statistical analyses

Group differences in demographic and substance use variables were analyzed using independent *t*-tests or ANOVAs, as applicable. Frequency data were evaluated by means of Pearson's chi-square tests. Group differences in neuroanatomical parameters (CT, CSA, and subcortical volume) and in cognitive performance scores at baseline were conducted by analyses of covariance (ANCOVA). Using Pearson's product-moment correlation analyses, associations between neuroanatomical measurements that showed a significant group effect at baseline and cocaine consumption parameters were conducted in the CU group. As we had the a priori-hypothesis that cortical structures are negatively associated with duration of cocaine use, cumulative dose of cocaine, grams consumed per week and positively with age of onset, one-tailed correlations were conducted. Correlations including the duration and the cumulative dose of cocaine consumption were adjusted for age because of the strong link between those variables and age. To investigate if there is a link between alterations in cortical neuroanatomical parameters and cognitive ability, we additionally analyzed the relation between measures for CT/CSA and cognitive outcome scores at baseline (one-tailed).

For longitudinal evaluation mixed design ANCOVAs including controls, *Decreasers*, and *Sustained Users*, were used. Sidak-corrected pairwise pre-post comparisons and follow-up group comparisons were conducted after a significant omnibus test (group ∗ time). To correct for cognitive test-retest effects, the CU change scores were adjusted by subtracting the controls mean change scores, as controls performance over the study period was assumed to be constant. To relate neuroanatomical changes found to cocaine use during the test interval, correlations were conducted between the measured change in neuroanatomical structures and the cumulative amount of cocaine consumed between the two measurement points in the CU groups. Similar to the baseline analyses, we additionally linked cognitive change scores with possible changes found in CT/CSA and subcortical volumes.

All ANCOVAs (cross-sectional and longitudinal) were adjusted for ADHD-SR score and IQ scores, two variables that have formerly been linked to neuroanatomical alterations, cognitive performance and substance consumption in general (Preller et al., 2014; Proal et al., 2011; Schnack et al., 2015; Vonmoos et al., 2013). Group comparisons at baseline were additionally corrected for age, a recommended covariate in MRI research (Barnes et al., 2010). Compared to baseline, *Sustained Users* consumed significantly more grams of alcohol per week than the *Decreasers* at follow-up (F_1,27_ = 6.64, P = .016, d = 0.87). Thus, longitudinal ANCOVAs were additionally adjusted for the amount of alcohol consumed between baseline and follow-up, and the time between the two measurements. All analyses including either CSA or subcortical volumes, total surface area or ICV were additionally included as a covariate, respectively. Moreover, except for duration of cocaine use and age of onset, the consumption variables were not normally distributed and were therefore ln-transformed for correlation analyses. The level of significance for all tests was set to P < .05. Additionally, a false discovery rate (FDR) correction was used for cross-sectional group comparisons for each modality and time point separately (significant findings are indicated with an asterisk, Benjamini and Hochberg, 1995). All statistical analyses were performed using IBM SPSS Statistics 22.0 (SPSS Inc.) for Mac.

## 3 Results

### 3.1 Demographic characteristics

Controls and both CU groups were matched for age, gender, and time between the two measurements (Table 1). Similar to former studies including CU and controls, *Decreasers* and *Sustained Users* differed from the control group in the subjective rating of ADHD symptoms, and BDI score (Vonmoos et al., 2013, Vonmoos et al., 2014). *Sustained Users* had a significantly lower verbal IQ than the controls and *Decreasers*. At baseline both user groups showed similar cocaine consumption severity indicated by the lack of group differences in subjective as well as objective (hair concentrations) measures. In contrast, a ten-fold higher cocaine concentration in hair samples was found in *Sustained Users* compared to *Decreasers* at follow-up. Furthermore, the cocaine_total_ concentration and subjectively reported amount of cocaine used per week were strongly correlated at both time points (baseline: r(23) = 0.58, P = .0026; follow-up: r(22) = 0.63, P = .0012). Finally, at baseline *Decreasers* (80%) and *Sustained Users* (71%) did not differ regarding their frequency of a DSM-IV diagnosis of current cocaine dependence (χ^2^ = 0.29, P = .590). However, more *Sustained Users* (79%) than *Decreasers* (33%) were currently dependent at the follow-up (χ^2^ = 5.99, P = .014). At the follow-up four *Decreasers* actually stopped cocaine intake for at least 6 months as confirmed by hair analyses.

**Table 1:** Demographic data and substance use information.

	*Baseline*							*Follow-up*						
	Controls (*n* = 38)	Decreasers (*n* = 15)	Sustained Users (*n* = 14)	*F*/χ^2^/*t*	*df, df*_*err*_	*P*	*Effect size*	Controls (*n* = 38)	Decreasers (*n* = 15)	Sustained Users (*n* = 14)	*F*/χ^2^/*t*	*df, df*_*err*_	*P*	*Effect size*
Age (year)	30.0 (6.8)	31.5 (5.5)	32.9 (7.1)	1.08^a^	2,64	0.34	_p_η^2^ = 0.03							
Sex (female/male)	15/23	7/8	2/12	3.81^b^	2	0.15	V = 0.24							
Hair color (bl/br/blk)^d^	0/31/3	0/11/3	0/10/1	1.61^b^	2	0.49	V = 0.17							
Years between TP1/TP2	1.5 (1.3)	1.8 (1.6)	1.6 (1.5)	0.32^a^	2,64	0.73	_p_η^2^ = 0.01							
Education (year)	10.5 (1.5)	10.7 (1.7)°	9.4 (0.7)^⁎^	3.99^a^	2,64	**0.02**	_p_η^2^ = 0.11							
Verbal IQ (MWT-B)	107.2 (9.8)	104.5 (11.8)	98.2 (9.0)^⁎^	4.02^a^	2,64	**0.02**	_p_η^2^ = 0.11							
ADHD-SR score	6.7 (5.7)	13.8 (8.6)^⁎⁎^	18.8 (9.0)^⁎⁎⁎^	16.24^a^	2,64	**<0.001**	_p_η^2^ = 0.34							
BDI score	2.6 (3.0)	8.2 (8.2)^⁎⁎^	10.2 (9.5)^⁎⁎⁎^	9.74^a^	2,64	**<0.001**	_p_η^2^ = 0.23	2.4 (4.7)	5.7 (5.4)	10.5 (11.4)^⁎⁎^	7.59^a^	2,64	**0.001**	_p_η^2^ = 0.19
Cocaine														
Times per week^e^	–	2.4 (2.0) n = 14	2.7 (1.9) *n* = 13	0.44^c^	25	0.66	d = 0.17	–	0.6 (0.9)	2.6 (2.2)	3.09^c^	27	**0.007**	d = 1.27
Grams per week^e^	–	1.9 (1.4)	5.9 (13.5)	1.15^c^	27	0.26	d = 0.54	–	0.7 (1.3)	4.7 (8.8)	1.68^c^	27	0.12	d = 0.79
Years of use	–	7.5 (5.6)	8.6 (5.2)	0.56^c^	27	0.58	d = 0.21	–	9.0 (6.2)	10.2 (6.2)	0.54^c^	27	0.60	d = 0.20
Cumulative dose (g)^f^	–	811 (929)	1966 (2125)	1.87^c^	27	0.08	d = 0.76	–	80 (90) n = 14	290 (696)	1.12^c^	26	0.27	d = 0.53
Maximum dose (g/d)	–	4.5 (3.0)	3.9 (3.5)	0.47^c^	27	0.64	d = 0.18	–	2.2 (1.7) *n* = 10	3.7 (4.6) *n* = 12	0.94^c^	20	0.36	d = 0.46
Last consumption (d)	–	10 (15) n = 14^i^	10 (11)	0.11^c^	26	0.92	d = 0.04	–	54 (110) n = 13^k^	6 (5)	1.57^c^	25	0.14	d = 0.84
Cocaine craving	–	30.1 (15.7) n = 14	26.1 (11.8) n = 13	0.76^c^	25	0.46	d = 0.30	–	15.5 (6.7)	29.4 (17.2) n = 13	2.75^c^	26	**0.02**	d = 1.17
Hair analysis (ng/mg)^d^														
Cocaine_total_	–	13.3 (15.6)	27.7 (40.6)	1.23^c^	23	0.23	d = 0.51	–	4.9 (6.0)	52.2 (37.8)	4.46^c^	26	**0.001**	d = 2.15
Cocaine	–	9.9 (11.1)	21.0 (30.9)	1.25^c^	23	0.22	d = 0.53	–	3.7 (4.6)	37.8 (28.4)	4.28^c^	26	**0.001**	d = 2.07
Benzoylecgonine	–	3.1 (4.3)	6.2 (9.2)	1.12^c^	23	0.28	d = 0.46	–	1.1 (1.5)	13.5 (11.0)	4.02^c^	26	**0.002**	d = 1.99
Cocaethylene	–	0.6 (1.0)	0.6 (0.6)	0.14^c^	23	0.89	d = 0.06	–	0.5 (0.6)	1.5 (1.7)	2.12^c^	26	0.05	d = 0.91
Norcocaine	–	0.3 (0.4)	0.5 (0.6)	1.04^c^	23	0.31	d = 0.42	–	0.1 (0.1)	0.9 (0.6)	4.59^c^	26	**<0.001**	d = 2.09
Urine toxicology (neg/pos)^g^	38/0	8/7	7/7	0.03^b^	1	0.86	V = 0.03	38/0	11/4	6/8	2.77^b^	1	0.10	V = 0.31
Alcohol														
Grams per week^e^	77.9 (110.7)	265.0 (598.4)	306.2 (607.0)	2.24^a^	2,64	0.12	_p_η^2^ = 0.07	61.2 (69.2)	135.5 (131.2)°	333.6 (414.3)^⁎⁎⁎^	9.17^a^	2,64	**<0.001**	_p_η^2^ = 0.22
Years of use	11.0 (6.2) *n* = 35	12.3 (7.7)	13.4 (6.9)	0.65^a^	2,61	0.53	_p_η^2^ = 0.02	12.4 (6.6) n = 35	14.1 (8.5)	15.0 (7.4)	0.78^a^	2,61	0.46	_p_η^2^ = 0.03
Nicotine														
Smoking (yes/no)	27/11	12/3	14/0	5.20^b^	2	0.07	V = 0.28	28/10	12/3	14/0	4.54^b^	2	0.10	V = 0.26
Cigarettes per day^e^	5.0 (6.2)	9.9 (11.3)	12.5 (8.1)^⁎^	5.33^a^	2,64	**0.007**	_p_η^2^ = 0.14	4.7 (6.4)	10.3 (14.2)	11.8 (7.5)^⁎^	4.32^a^	2,64	**0.02**	_p_η^2^ = 0.12
Years of use	7.1 (6.9)	9.1 (8.4)	14.5 (6.1)^⁎⁎^	5.42^a^	2,64	**0.007**	_p_η^2^ = 0.15	8.1 (7.5)	10.6 (9.3)	16.1 (6.7)^⁎⁎^	5.46^a^	2,64	**0.006**	_p_η^2^ = 0.15
Cannabis														
Grams per week^e^	0.0 (0.2)	2.3 (7.2)	2.4 (5.0)	2.59^a^	2,64	0.08	_p_η^2^ = 0.08	0.1 (0.2)	0.2 (0.5)°	1.5 (2.4) ^⁎⁎^	8.03^a^	2,64	**0.001**	_p_η^2^ = 0.20
Years of use	3.7 (5.6) *n* = 37	7.9 (8.7)	12.9 (6.6)^⁎⁎⁎^	10.25^a^	2,63	**<0.001**	_p_η^2^ = 0.25	3.9 (5.8) n = 37	8.9 (9.9)	13.6 (7.1)^⁎⁎⁎^	9.89^a^	2, 63	**<0.001**	_p_η^2^ = 0.24
Cumulative dose (g)	73 (228) n = 37	1719 (2226)	3324 (4519)^⁎⁎⁎^	10.66^a^	2,63	**<0.001**	_p_η^2^ = 0.25	1.8 (7.1) n = 37	30.0 (53.6)	50.5 (70.3)^⁎⁎^	8.11^a^	2,64	**0.001**	_p_η^2^ = 0 0.20
Last consumption (d)^h^	58 (61) n = 10^i^	15 (20) *n* = 8^k^	22 (17) *n* = 9	3.24^a^	2,24	0.06	_p_η^2^ = 0.21	69 (63) *n* = 11	21 (36)^⁎^ n = 10	11 (11)^⁎⁎^ n = 12	6.00^a^	2,30	0.006	_p_η^2^ = 0.29
Urine toxicology (neg/pos)^g^	37/1	11/4	11/3	7.42^b^	2	**0.02**	V = 0.33	37/1	12/3	11/3	5.75^b^	2	0.06	V = 0.29
Amphetamine														
Grams per week^e^	0.0 (0.0)	0.3 (0.5)^⁎⁎^° n = 14	0.0 (0.1)	6.16^a^	2,63	**0.004**	_p_η^2^ = 0.16	0.0 (0.0)	0.0 (0.0)°	0.1 (0.2)^⁎⁎^	7.28^a^	2,64	**0.001**	_p_η^2^ = 0.19
Years of use	0.0 (0.0)	4.0 (4.2)^⁎⁎⁎^	2.1 (2.9)^⁎^ n = 13	16.46^a^	2,63	**<0.001**	_p_η^2^ = 0.34	0.1 (0.5)	4.4 (4.4)^⁎⁎⁎^	2.4 (3.0)^⁎^ n = 13	17.05^a^	2,63	**<0.001**	_p_η^2^ = 0.35
Cumulative dose (g)	0 (0)	75 (128)	154 (307)^⁎⁎^	5.63^a^	2,64	**0.006**	_p_η^2^ = 0.15	0.1 (0.8)	9.1 (31.8)	3.7 (5.5)	1.93^a^	2, 65	0.16	_p_η^2^ = 0.06
Last consumption (d)^h^	–	27.5 (24.9) n = 8	67.7 (70.8) n = 5	2.25^a^	1,11	0.16	_p_η^2^ = 0.17	30 (−) n = 1	49 (45) *n* = 7	37 (48) n = 7	0.16^a^	2,12	0.86	_p_η^2^ = 0.03
Hair analysis (ng/mg)^d^	0.0 (0.0)	1.2 (3.3)	0.6 (1.4)	2.42^a^	2,57	0.10	_p_η^2^ = 0.08	0.0 (0.0)	0.2 (0.4)	0.5 (1.3)	3.06^a^	2,62	0.05	_p_η^2^ = 0.09
Urine toxicology (neg/pos)^g^	38/0	15/0	15/0	–	–	–	–	38/0	14/1	13/1	2.71^b^	2	0.26	V = 0.20
MDMA														
Tablets per week^e^	0.0 (0.0)	0.2 (0.4)	0.2 (0.5)	2.58^a^	2,64	0.08	_p_η^2^ = 0.07	0.0 (0.0)	0.1 (0.2)°° n = 14	0.8 (1.2)^⁎⁎⁎^	11.55^a^	2,63	**<0.001**	_p_η^2^ = 0.27
Years of use	0.1 (0.6)	4.7 (5.4)^⁎⁎⁎^	3.9 (4.8)^⁎⁎^ n = 13	13.32^a^	2,63	**<0.001**	_p_η^2^ = 0.30	0.2 (1.1)	5.3 (6.1)^⁎⁎⁎^	4.6 (5.0)^⁎⁎^ n = 13	13.46^a^	2,64	**<0.001**	_p_η^2^ = 0.30
Cumulative dose (tablet)	0 (2)	107 (210)°	598 (1011)^⁎⁎⁎^ n = 13	8.52^a^	2,63	**0.001**	_p_η^2^ = 0.21	0.6 (3.5)	4.0 (8.6)°°°	30.6 (36.0)^⁎⁎⁎^	16.54^a^	2,64	**<0.001**	_p_η^2^ = 0.34
Last consumption (d)^h^	–	51.0 (44.4) n = 6	78.2 (67.4) n = 7	0.71^a^	1,11	**0.42**	_p_η^2^ = 0.06	91 (−) n = 1	34 (18) n = 7	39 (55) n = 10	0.74^a^	2,15	0.49	_p_η^2^ = 0.09
Hair analysis (ng/mg)^d^	0.0 (0.0)	0.2 (0.3)°	1.2 (2.3)^⁎⁎^	6.52^a^	2,57	**0.003**	_p_η^2^ = 0.19	0.0 (0.0)	0.2 (0.3)	1.2 (3.3)^⁎^	3.59^a^	2,63	**0.03**	_p_η^2^ = 0.10
Methylphenidate														
Cumulative dose (tablet)	3 (20)	70 (262)	214 (524)^⁎^ n = 13	3.20^a^	2,63	**0.05**	_p_η^2^ = 0.09	0.00 (0.00)	28.4 (106.4) n = 14	20.7 (70.7)	1.51^a^	2,62	0.23	_p_η^2^ = 0.05
Hair analysis (ng/mg)^d^	0.0 (0.0) *n* = 30	0.0 (0.0) n = 12	0.0 (0.0) n = 10	1.65^a^	2,49	0.20	_p_η^2^ = 0.06	0.0 (0.0)	0.1 (0.3)	0.0 (0.0)	1.70^a^	2,63	0.19	_p_η^2^ = 0.05

Means and SD of all groups (controls, decreasers and sustained users) for both measurement time points.

Significant P values are shown in bold.

Abbreviations: ADHD-SR = ADHD self-rating scale; BDI=Beck Depression Inventory; bl = blonde; blk = black; br = brown; d = day; g = gram; MDMA = 3,4methylenedioxymethamphetamine (“ecstasy”); mg = milligram; MWT-B = Mehrfachwahl-Wortschatz-Intelligenztest; ng = nanogram.

a ANVOA group comparisons with significant Sidak post-hoc tests (cocaine user groups vs. controls: *P < .05, **P < .01,***P < .001; Decreasers vs. Sustained Users: °P < .05, °°P < .01,°°°P < .001.

b χ^2^-test for frequency data (cocaine user groups only/all groups); Kramer's V reported as effect-size.

c *t*-test (independent, two-tailed, cocaine users only); Cohen's d reported as effect-size.

d Hair samples for four controls and four cocaine users are missing at baseline. At follow-up, hair sample for one sustained user is missing.

e Average of use over the last six months.

f At baseline the total cumulative dose across lifetime and at follow-up the total dose of cocaine consumed between the two measurement points are meant.

g Urine tests classifications (neg/pos) are based on the following cutoff values: amphetamine = 300 ng/ml; cocaine = 150 ng/ml; tetrahydrocannabinol = 50 ng/ml (Substance Abuse and Mental Health Services Administration, 2008). The frequency tests for amphetamine and cannabis included all three groups, whereas for cocaine only the two cocaine user groups.

h Days passed until last consumption were averaged only for subjects using the substance within the last six months.

i Data for one participant is missing.

k Data for 2 participants are missing.

### 3.2 Baseline group differences in neuroanatomical parameters and cognition

At baseline, significant group effects were found for CT in the SFG, and IFG as well as in the lateral OFC (F_1,62_ = 5.59–6.83, P = .021–0.11; Fig. 1A and Suppl. Table 1). In all of the analyzed ROI, thinner cortices were found in CU compared to controls. Group differences were additionally found in the ACC where CU showed a significantly smaller CSA than controls (Fig. 1B and Suppl. Table 1). For subcortical structures, we found no significant differences after adjustment for multiple comparisons (Fig. 2, Suppl. Table 1). All results remained the same when gender was included as an additional covariate (Suppl. Table 2). Moreover, ADHD-SR score did not significantly explain any variance in the baseline analyses for CT and CSA. However, the ADHD-SR score did significantly explain variance in the model investigating the globus pallidus volume difference between the groups (P = .003) but not for other subcortical structures. Moreover, the two CU groups did not differ in any measured GM variables (CT, CSA, subcortical volumes) at baseline (Suppl. Table 3).

In line with the literature, CU scored significantly lower in tests measuring sustained attention (controls mean = 0.92 SE = 0.01, CU mean = 0.89 SE = 0.01; F_1,47_ = 7.12, P = .010, d = 0.80) and working memory (controls mean = 16.6 SE = 0.6, CU mea*n* = 13.8 SE = 0.7; F_1,59_ = 8.44, P = .005, d = 0.82) compared to controls.

### 3.3 Baseline associations between neuroanatomical parameters, cocaine use, and cognition

To evaluate the association between cocaine use parameters and cortical measurements, we only analyzed ROI and subcortical structures where significant group differences were found. Additionally, as all significant regions of CT (i.e., SFG, IFG, and lateral OFC) showed the same pattern of reduction, those ROI were further averaged to one region in order to avoid the problem of alpha-error accumulation. Significant associations were found between the average CT of the three significant ROI and the cumulative lifetime consumption (in g) (r(26) = −0.33, P = .044) and the duration of cocaine use (r(26) = −0.35, P = .036; Suppl. Table 4). Specifically, the larger the amount of cocaine used and the longer cocaine had been used, the thinner the lateral frontal regions were (Suppl. Fig. 3A and B). No associations were found for the CSA (ACC). Additionally, correlations with other substances did not reach significance and are reported in Suppl. Table 5. Importantly, the amount of cigarettes smoked a day was not significantly correlated with alterations seen in CT, and CSA (Suppl. Table 5) supporting earlier findings that show that frontal GM alterations found in CU are not associated with smoking severity (Crunelle et al., 2014).

**Fig. 3:**
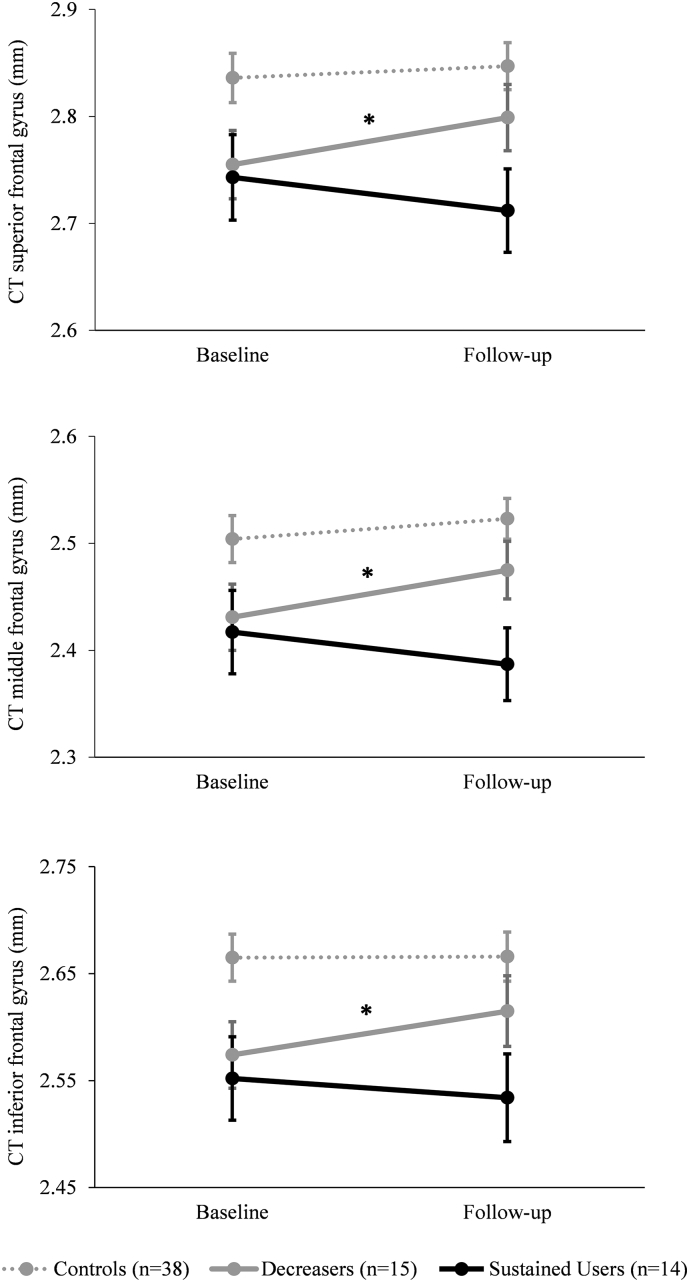
Changes in cortical thickness. Change in cortical thickness (in mm) from baseline to follow-up. Superior frontal gyrus: *Decreasers* P = .018 *Sustained Users* P = .16; Middle frontal gyrus: *Decreasers* P = .009 *Sustained Users* P = .15; Inferior frontal gyrus: *Decreasers* P = .022 *Sustained Users* P = .39; Sidak post-hoc tests: *P < .05. All analyses were corrected for verbal IQ, ADHD-SR score, the amount of alcohol consumed between baseline and follow-up, and the time interval between the two measurements.

There was a significant association between CT and sustained attention (r(21) = 0.36, P = .048) but the correlation with working memory was not significant (r(24) = −0.22, P = .15). Moreover, correlation analyses between CSA (ACC) and both cognitive measures were not significant (RVP: r(21) = 0.26, P = .12; LNST: r(24) = 0.14, P = .25).

### 3.4 Longitudinal changes in neuroanatomical parameters and cognition

Applying mixed ANCOVAs significant group ∗ time interactions were found for CT in the SFG (F_2,60_ = 4.16, P = .020, _p_η^2^ = 0.12), MFG (F_2,60_ = 4.64, P = .013, _p_η^2^ = 0.13), and the IFG (F_2,60_ = 3.23, P = .047, _p_η^2^ = 0.10; Fig. 3). For CT within the medial, lateral OFC, and ACC no significant interactions were found (F_2,60_ = 1.23–2.88, P = .06–0.30, _p_η^2^ = 0.04–0.09). A lack of significant interaction effects was also apparent in all ROI for CSA (F_2,59_ = 0.31–3.06, P = .05–0.96, _p_η^2^ = 0.001–0.09) and subcortical structures (F_2,59_ = 0.02–1.86, P = .16–0.98, _p_η^2^ = 0.001–0.06). The results remained the same when the cumulative amounts of cannabis consumption or tobacco smoking (cigarettes per day at follow-up) were added as additional covariates in separate ANCOVAs. Subsequent Sidak-corrected tests for group comparisons at follow-up revealed that CT was significantly thicker in controls compared to *Sustained Users* in the SFG (P = .026), MFG (P = .008), and IFG (P = .047). There were no group differences found between the controls and *Decreasers* at follow-up (P = .43–0.56). Pairwise Sidak pre-post comparisons additionally showed that *Decreasers* significantly increased their CT within all three regions where significant omnibus tests (group*time) were found (SFG: P = .018; MFG: P = .009; IFG: P = .022). Within the same regions, CT in *Sustained Users* seemed to further decrease over time, but this pre-post comparison did not reach significance (P = .15–0.39).

**Fig. 4:**
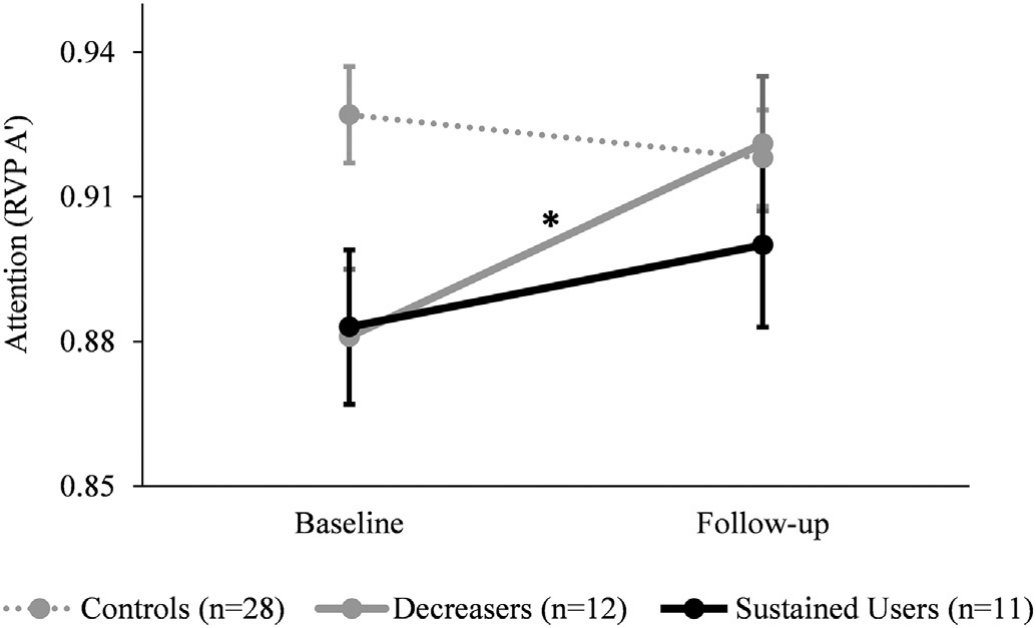
Changes in attention performance. Change in sustained attention performance from baseline to follow-up. The analysis was corrected for verbal IQ, ADHD-SR score, the amount of alcohol consumed between baseline and follow-up, and the time interval between the two measurements. The follow-up values were additionally adjusted for test-retest effects. *Decreasers* P = .00003 *Sustained Users* P = .11; Sidak post-hoc tests: *P < .05.

The results for the two cognitive measures revealed a significant group ∗ time interaction for sustained attention (F_2,44_ = 9.07, P = .0005, _p_η^2^ = 0.29; Fig. 4), but not for working memory (F_2,56_ = 1.32, P = .27, _p_η^2^ = 0.05). Subsequent post-hoc analyses for RVP showed that there were no significant group comparisons applicable at follow-up (controls vs. *Decreasers*: P = 1.0; controls vs. *Sustained Users*: P = .81). However, pairwise Sidak pre-post comparisons showed that the *Decreasers* had a performance improvement when comparing performance scores at baseline vs. follow-up (P = .00003).

### 3.5 Associations between changes in neuroanatomical parameters, cocaine consumption, and cognition

Again, only regions where a significant time ∗ group omnibus test was found were further analyzed. These three ROI were again averaged to one region in order to reduce the risk of multiple comparisons. Thickness change in this merged region was significantly associated with the cumulative amount of cocaine used within the time interval (r(26) = −0.32, P = .047; Suppl. Fig. 4). The CT change of this region was not associated with other illicit substances/alcohol used during the study period (Suppl. Table 6). Moreover, CT changes was also not associated with ADHD-SR scores neither in the entire sample (r(65) = −0.11, P = .19, 1-tailed) nor when we restricted our calculations to the CU groups (r(27) = −0.03, P = .44, 1-tailed). Regrettably, for smoking we do not have reliable data for cumulative use of cigarettes over the study interval. However, the amount of cigarettes smoked per day at follow-up did not correlate significantly with changes seen in CT (r(27) = 0.05, P = .40, 1-tailed).

Furthermore, the change in CT was positively linked to attention performance within the CU group (r(21) = 0.36, P = .044; Suppl. Fig. 5), but not to working memory (r(24) = 0.26, P = .10). Moreover, the relationship between CT change and attention performance remained significant when controlling for cocaine consumption within the interval (r(19) = 0.40, P = .035). In contrast, the aforementioned association between CT change and cumulative cocaine consumption did not remain significant when controlling for attention performance change (r(19) = −0.27, P = .12). However, the downshift of the latter correlation was not significant (from *r* = −0.32 to −0.27; z = 0.16, P = .44), so that we would not like to overinterpret this result.

## 4 Discussion

The main goal of this study was to investigate whether consistently reported GM alterations in CU might be substance-induced and potentially reversible. With a longitudinal study design including individuals decreasing or increasing their cocaine consumption, we anticipated getting valuable information about the possible plasticity of neuroanatomical structures. Moreover, we were interested to link our findings to changes in cognitive performance to further enhance our knowledge about potential neuroanatomical underpinnings of well-documented working memory and attention deficits in CU (Jovanovski et al., 2005; Vonmoos et al., 2013). Using a surface-based morphometry approach we investigated CT and CSA (instead of volumetric measures) of the cortex of CU and controls. Our main results showed CT recovery in lateral frontal regions after decreasing or ceasing cocaine intake. In contrast–albeit not significantly–CT further declined in *Sustained Users* in the same regions over the study period. These CT changes were additionally associated with the cumulative amount of cocaine consumed within the time interval and changes in attention performance scores. To our knowledge, this study is the first to show both, possible recovery but also further loss of CT within the frontal lobe in chronic CU. Moreover, CT changes might be the cause of cognitive difficulties seen in CU. Thus, our findings provide new target regions for possible therapeutic interventions.

### 4.1 Baseline associations between neuroanatomical parameters, cocaine use, and cognition

In a first step, we replicated former findings of apparent diminished frontal GM volume in CU (for a review see: Ersche et al., 2013b; Hanlon et al., 2011; Ide et al., 2014). We found lower CT in the SFG, IFG, and lateral OFC in CU compared to controls. CT within ACC, and the medial OFC was not significantly different between the two groups. This was unexpected, as alterations within medial frontal regions have been most consistently reported (Ersche et al., 2011; Franklin et al., 2002; Matochik et al., 2003). Interestingly, we found significant smaller ACC surface areas in CU compared to controls, while no significant CSA differences were found in all other ROI (d = 0.02–0.29). Although both cortical measures (CT and CSA) are reported to be heritable, they seem to be genetically unrelated (Panizzon et al., 2009; Winkler et al., 2010). Moreover, the two structural properties are also suggested to represent different facets of the cortex according to the radial unit hypothesis (Rakic, 1988, Rakic, 1995): Whereas the size of CSA is believed to be related to the number of cortical columns, CT is related to the number, size, and packing density of cells within such a column (Rakic, 1988). Our results indicate that medial and lateral frontal lobe GM abnormalities might be the result of different underlying mechanisms. Thus, GM alterations within lateral areas might be due to either the reduction of cell volume, cell death, and loss of dendritic branching or a combination of these possibilities. In contrast, the CSA differences seen within the ACC could be more related to a lower amount of cortical columns in this area. In general, our findings highlight the importance of analyzing these two independent variables of cortical volume separately.

With respect to subcortical structures, we found no differences between CU and healthy controls after correction for multiple comparisons. However, we would like to point out that the group difference regarding thalamus volume reached at least a medium effect size (d = 0.46). Compared to consistently shown cortical abnormalities, results regarding subcortical structures in CU are partially inconsistent: while smaller thalamus volumes within CU have been confirmed by a meta-analysis (Ersche et al., 2013b), results for striatum abnormalities are rather contradicting. Larger (Ersche et al., 2011, Ersche et al., 2012) as well as smaller striatum volumes (Barros-Loscertales et al., 2011; Schuch-Goi et al., 2017) in addition to lack of such a difference have been reported (Ersche et al., 2013b; Garza-Villarreal et al., 2016). These discrepancies could be related to methodological reasons such as sample characteristics, but also to variations in sample size, as the effects on thalamic and striatal structures might be subtler and more participants are needed to indicate subcortical alterations compared to cortical GM differences (Mackey and Paulus, 2013) (see also below). In addition, discrepancies could also emerge from different covariates used (e.g., age, ADHD symptoms). Moreover, in this study, an automatic segmentation algorithm was employed. Manual segmentation, when performed by a well-trained individual, has been shown to be more reliable than automated segmentation. However, manual segmentation is highly time consuming – specifically in large MRI data sets, such as ours, therefore simply not feasible – and can introduce biases and errors (Boccardi et al., 2011). In contrast, automated segmentation techniques have been shown to achieve reliable and reproducible segmentations of brain areas and have demonstrated the ability to capture brain changes associated with disease (Chakravarty et al., 2013; Fischl et al., 2002; Makowski et al., 2018).

As hypothesized, we also found reduced cognitive performance levels in CU compared to controls within both cognitive domains tested. This stands in agreement with earlier studies reporting pronounced deficits in dependent but also recreational users' attention and working memory performance (Jovanovski et al., 2005; Vonmoos et al., 2013). Moreover, we found a relationship between the CU's sustained attention deficits and lower frontal CT. Worse attention performance seen in CU might therefore be due to thinning within the lateral frontal lobe in CU. Additionally, we investigated the association between CT and consumption variables and found the higher the cumulative consumed cocaine amount was, and the longer cocaine has been used, the thinner the lateral frontal cortex regions were. Duration of cocaine intake was formerly linked to diminished GM volume within similar regions as in our study (e.g., SFG, MFG, IFG; Barros-Loscertales et al., 2011; Lim et al., 2008).

### 4.2 Longitudinal changes in neuroanatomical parameters and cognition

#### 4.2.1 Regional specificity in cortical thickness changes

The longitudinal analyses conducted in the second step of this study strengthen the notion of possible cocaine-induced alterations within the frontal cortex. Our findings indicate that decreasing cocaine consumption was associated with increasing CT at follow-up. Such a GM recovery was also previously described in another longitudinal study (Parvaz et al., 2017). Investigating GM changes in abstinent CU the authors found recovery in GM volumes in the IFG and ventromedial prefrontal regions (Parvaz et al., 2017). The lack of ventromedial prefrontal recovery in our study might be due to the fact that 11 of the 15 *Decreasers* were still using cocaine. Thus, our data suggest that ventromedial prefrontal regions might be more sensitive to cocaine exposure and only recover when intake is completely ceased. In addition, also former cross-sectional data from different abstainer groups depicted a positive association between days of abstinence and GM volume (Hanlon et al., 2011). However, to the best of our knowledge, this is the first study that longitudinally investigates changes in groups of CU with decreased and sustained cocaine intake using hair testing as an objective determination of cocaine use. In contrast to participants who decreased their consumption, subjects with increased or sustained high use of cocaine showed a tendency of further decreased frontal CT. The lack of significance for this CT change in *Sustained Users* is not surprising as they already reported a relatively long duration of cocaine use with a mean of eight years at baseline. They had a cumulative lifetime intake of about 2 kg cocaine, thus, the additional 290 g that they had consumed in average over the study interval was possibly too small to cause another significant decline.

#### 4.2.2 Possible mechanisms for longitudinal cortical thickness changes

Next to a significant CT recovery in *Decreasers*, CT seemed to further decline with ongoing use of cocaine. This additional CT decline with sustained cocaine use would support the notion of an ongoing neuroplastic or neurotoxic effect. This suggestion is further supported by a study that found sustained smaller frontal GM volume in CU who were abstinent for 5 years (Tanabe et al., 2009). Similarly, *Decreasers* had still lower CT than controls at follow-up. However, due to the lack of existing longitudinal data, it is not known how long a possible full GM recovery would take; thus, the interval in our study could have been too short. Moreover, GM patterns of recovery found in our study but also reported by Parvaz et al. (2017) stand against a predominantly neurotoxic effect. Thus, the changes might rather be a combination of toxicity and maladaptation through neuroplasticity.

Therefore, the GM changes reported in the literature and found in this study could be linked to several underlying mechanisms. One possible explanation for GM reduction is the notion of cocaine-induced oxidative stress due to excessive dopamine (DA) accumulation (Pereira et al., 2015). The involvement of the DA system in alterations seen in the frontal cortex is likely, as this region is the major cortical target area of DA afferents (Tritsch and Sabatini, 2012). It has been shown that cocaine acutely increases extracellular dopamine in the prefrontal cortex (Pum et al., 2007; Saunders et al., 1994). The subsequent increased metabolism due to DA accumulation leads to a buildup of reactive oxygen species within the synaptic cleft, leading to oxidative stress and posterior to axon terminal degeneration and possible cell death (Jang et al., 2015; Pereira et al., 2015). The idea of a DA involvement in the alterations seen is further supported by the positive link between the prefrontal cortex's basic metabolic activity and DA receptor depletion within subcortical regions (Volkow et al., 1993). Further, imbalance of DA within the frontal cortex is also associated with cognitive performance deficits in working memory and attention (Clark and Noudoost, 2014; Tritsch and Sabatini, 2012). With a positive relationship found between CT and cognitive performance in our data, DA-related maladjustments, leading to abnormalities in GM, might be the neuroanatomical underpinning of cognitive deficits. Alternative explanations for GM change could also involve other neurotransmitter systems such as GABA, as proposed by Parvaz et al. (2017). But also neuroplastic effects of the noradrenaline system might be linked to the changes seen in GM (Havranek et al., 2017; Preller et al., 2013; Ramos and Arnsten, 2007).

Moreover, cortical thinning, especially in frontal regions, is not unique to addiction. The pronounced reduction of GM volume of the frontal lobe is a common finding in aging literature (e.g., Lemaitre et al., 2012; Raz et al., 1997). Thus, the notion arose that the reported alterations found in GM could be indicative of an early aging brain status in CU (Ersche et al., 2013a). The life-style of addicted individuals is often linked to chronic stress, risky health-related behavior but also disturbed sleep patterns, all factors which might lead to an accelerated aging process (Bachi et al., 2017). In our study, we found changes especially in CT, which were additionally associated with attention performance changes. Similarly, recovery in the IFG was previously linked to improvement in cognitive flexibility (Parvaz et al., 2017). Interestingly, a recent study showed that the mean annual percentage change of GM in elderly subjects was higher in CT than compared to CSA (Storsve et al., 2014). Importantly, those frontal lobe deficits in aging are also linked to cognitive performance (Zimmerman et al., 2006).

#### 4.2.3 Possible reasons for the lack of cortical surface area and subcortical volume changes

The lack of change for CSA is interesting as we found that CSA for the ACC to be smaller in CU at baseline compared to controls. Reasons for the absence of change could again be manifold: First, the GM abnormalities in CSA seen at baseline could depict non-reversible neurotoxic effects. However, we did not find a linear relationship with drug use parameters. Thus, if there was a neurotoxic effect, this effect might at least not be dose-dependent. Second, changes in CSA may follow a different time trajectory for recovery than CT. Third, CSA differences seen at baseline could represent a pre-existing state, i.e., participants with lower CSA in ACC might be more prone to start using cocaine but without a dose-dependent relation.

Regarding subcortical volumes we found neither a difference at baseline between CU and controls nor a change in subcortical volumes over the study interval. This could indicate that cocaine has either no chronic effect on the volume of subcortical structures or that the effects of cocaine on these structures are so weak that they could not be detected with our medium-powered study sample. In general, more and larger longitudinal studies investigating subcortical volumes as well as CSA are needed to answer these open questions. Nevertheless, in regard to CSA our results again highlight the importance of not only analyzing GM volume, but also CT and CSA separately.

### 4.3 Limitations

To the best of our knowledge, this is the first study that longitudinally investigated subjects with decreased but also individuals with sustained cocaine use including MRI data and objective measurements of cocaine use by hair testing. Nonetheless, the present results should be interpreted with the following limitations in mind. First, our study sample consisted of subjects using cocaine as primary drug (confirmed by quantitative hair analyses). Moreover, participants were already using cocaine at the first measurement time point, which might limit the interpretability regarding preexisting vulnerability markers. Furthermore, the time interval between the two measurement points ranged from 6 to 53 months. Although the groups were matched for follow-up interval and we added the interval duration as a covariate in our longitudinal analyses, we cannot completely rule out that high interval variability might have influenced our results. However, as we only have two time points available and no other study has ever reported data from more than two time points, we can only speculate about the impact the variability of the interval has. This is accentuated by the fact that at the moment we do not know the time window of GM recovery or the trajectory of GM change (e.g., is the change linear or non-linear). Maybe, participants with a longer period of abstinence (or reduction), recovered more as they had more time to do so. As stated above, Parvaz et al., (2017) showed significant recovery after six months, suggesting that the first months might be more crucial than the following months. The notion of a nonlinear GM recovery is supported by findings in alcohol research. Longitudinal data of GM volume changes of the frontal cortex in alcohol dependent subjects over three time points showed that the monthly rates of GM volume increases were larger between one week and one month compared to the longer interval between one and seven months (Durazzo et al., 2015; Zou et al., 2018). However, generalizations over different substances are questionable. Thus, further longitudinal studies (with >2 time points) are crucially needed to give some insight into this question. Lastly, although we tried to include only subjects with little co-use of other substances, exposure to other substances is nearly inevitable in most CU. Thus, we cannot completely dismiss effects of other substances. However, additional correlations at baseline and follow-up between other illicit drugs/alcohol consumption and GM alterations were not significant (Suppl. Table 6 and 7). Finally, we did not control for the potential impact of adulterants such as levamisole. A recent study from our laboratory showed that levamisole-exposure was associated with alterations in fronto-cortical structures of CU (Vonmoos et al., 2018). Unfortunately, in the present study levamisole was not determined in hair at baseline, thus, we were not able to analyze a possible relationship between changes in hair levamisole concentration and GM. The investigation of the influence of levamisole on brain structure in future longitudinal imaging studies with CU should be considered.

### 4.4 Conclusions

In summary, we found–beyond well-described frontal GM differences between CU and controls at baseline–neuroanatomical changes in frontal CT of CU depending on variations of their cocaine intake during the study interval. Our results showed that decreased or ceased cocaine intake lead to a significant thickening of the lateral frontal cortex, whereas sustained use was associated–albeit not significantly–with further thinning within the same region. Moreover, CT changes were additionally related to the amount of cocaine consumed during the follow-up interval, strengthening the suggestion that these changes are at least partially caused by cocaine intake. Further, CT changes were additionally associated with cognitive performance, leading to the notion that GM changes might be the neuroanatomical underpinnings of the cognitive deficits commonly found in CU. Thus, our results support the notion that especially frontal regions are potential target regions for psychotherapeutic and pharmacological interventions.

## Conflict of interest

The authors declare no conflict of interest.

## Appendix A Supplementary data

Supplementary material Image 1
