# Supplementary material for: Longitudinal changes in cocaine intake and cognition are linked to cortical thickness adaptations in cocaine users
Source: Neuroimage Clin. 2019 Jan 4;21:101652. doi: 10.1016/j.nicl.2019.101652 (PMC6412021; doi:10.1016/j.nicl.2019.101652)
Supplement: Supplementary file 1 — Supplementary material [file mmc1.docx]

**Supporting Information**

**Longitudinal changes in cocaine intake and cognition are linked to cortical thickness adaptations in cocaine users**

Sarah Hirsiger, PhD; Jürgen Hänggi, PhD; Jürgen Germann, MSc; Matthias Vonmoos, PhD; Katrin H. Preller, PhD; Etna J.E. Engeli, MSc; Matthias Kirschner, MD; Caroline Reinhard, MSc; Lea M. Hulka, PhD; Markus Baumgartner, PhD; Mallar M. Chakravarty, PhD; Erich Seifritz, MD; Marcus Herdener, MD; Boris B. Quednow, PhD

## Supplementary Methods 1. Recruitment and selection

Participants for the baseline measurement were recruited in the Zurich area with the help of associations involved in drug prevention (e.g., Streetwork Zurich), word-of-mouth advertising, newspaper and/or online advertisement, and from the Center of Addictive Disorders Zurich. All data was collected between 2010 and 2016 in the context of three independent cross-sectional imaging studies with cocaine users (CU) and healthy controls, which were all collected on the same scanner with exactly the same MRI-sequence (Engeli et al., in preparation; Kirschner et al., 2018; Preller et al., 2014). Participants had to be between 18 and 50 years of age at baseline, had to be able to provide written informed consent and to be fluent in German/Swiss German. Participants were excluded if they had a history of neurological disorders or head injuries, and suffered under severe somatic diseases. Additionally, subjects had to pass MRI safety requirements, prohibiting metallic implants, pacemaker, or neurostimulators.

Participants were re-invited at least 6 months after the first scan in the context of one of these cross-sectional studies. Because one of these studies was finished earlier (Preller et al., 2014) the time until the follow-up scan varied up to 53 months. Therefore, the test-retest interval was taken into account in the analyses. At follow-up 148 participants were contacted (76 controls, 72 CU) and data from 85 participants collected (42 controls, 43 CU). The drop-out rate of 40.3% for CU was expected and even lower than in a previous longitudinal study conducted in our laboratory (Vonmoos et al., 2014). Drop-out rate for controls was slightly higher with 44.7%. CU measured at baseline did not differ in age (t(70)=0.103, P=0.92), education (t(70)=-1.662, P=0.10), IQ (t(67)=0.599, P=0.55), ADHD-score (t(68)=1.497, P=0.139), cocaine grams per week (t(69)=0.390, P=0.70), years of cocaine use (t(69)=0.864, P=0.39), cumulative lifetime consumption of cocaine (t(69)=-0.109, P=0.91), and cocaine_total_ hair concentration (see below for further information) (t(62)=-1.443, P=0.16) from those who did not participate at follow-up. The same holds true for controls, participants measured were not different in age (t(74)=-0.068, P=0.95), education (t(74)=-0.032, P=0.98), IQ (t(73)=0.709, P=0.48), and ADHD-score (t(69)=0.062, P=0.95) from those who dropped out.
From the available 85 participants however, eighteen subjects (14 CU and 4 controls) had to be excluded. Five CU had to be excluded due to hair cocaine concentration below the cut-off value of 0.5 ng/mg. In addition, in two CU, hair analysis revealed that cocaine was not the primary used illicit drug. Furthermore, three CU were removed from the sample because all of them had either at baseline or at the follow-up measurement a positive urine test for opioids. Moreover, two CU strongly changed their medication over the study period (one participant started to use antipsychotic medication, one started the regular intake of venlafaxine at a high dose between the two measurement points). One CU had to be excluded due to an incidental pathological finding and one CU had to be excluded because of extensive head motion during the scan at the follow-up measurement. Unexpectedly, one control subject had to be excluded due to a positive urine sample for opioids, one due to cocaine traces in hair, and one due to regular escitalopram intake revealed by hair analysis. In addition, MRI data quality was not sufficient for one control subject. Leading to a final sample of 29 CU and 38 healthy controls. Four hair samples for controls were not available; those participants were nevertheless included in the analysis as their urine screening was negative and their answers in the drug interview were plausible and credible.

***Supplementary Methods 2.*** Urine and hair toxicology analyses

Urine toxicology analyses comprised the compounds/substances: tetrahydrocannabinol, cocaine, amphetamines, benzodiazepines, opioids, and methadone and were assessed by a semi-quantitative enzyme multiplied immunoassay method using a Dimension RXL Max (Siemens, Erlangen, Germany).

To characterize drug use exposure over the last months objectively, hair samples were collected at baseline and follow-up and analyzed with liquid chromatography-tandem mass spectrometry (LC-MS/MS). If participants’ hair was long enough, one sample of six cm hair (from the scalp) was taken from the occiput and subsequently divided into two subsamples of three cm length. After checking that both segments contained cocaine metabolites – because we were interested to measure only chronic CU – the values from both segments were averaged. At baseline and follow-up, 76% and 71.4% of CU had two segments available, respectively. Moreover, the average hair length for those CU with only one segment available was 2.60cm and 2.63cm at baseline and follow-up, respectively. The following compounds were assessed: cocaine, benzoylecgonine, ethylcocaine, norcocaine, levamisole, amphetamine, methamphetamine, MDMA, MDEA, MDA, ephedrine, morphine, codeine, dihydrocodeine, methadone EDDP (primary methadone metabolite), oxycodone, tramadol, methylphenidate and several medications.

For our routine protocol a three step washing procedure with water (2min shaking, 15ml), acetone (2min, 10ml) and finally hexane (2min, 10ml) of hair was performed. Then the hair samples were dried at ambient temperatures, cut into small snippets and extracted in two steps, first with methanol (5ml, 16h, ultrasonication) and a second step with 3ml MeOH acidified with 50µl hydrochloric acid 33% (3h, ultrasonication). The extracts were dried and the residue reconstituted with 50µl MeOH and 500µl 0.2mM ammonium formate (analytical grade) in water. As internal deuterated standards of the following compounds were used, added as mixture of the following compounds: cocaine-d3, benzoylecgonine-d3, ethylcocaine-d3, morphine-d3, MAM-d3, codeine-d3, dihydrocodeine-d3, amphetamine-d6, methamphetamine-d9, MDMA-d5. MDEA-d6, MDA-d5, methadone-d9, EDDP-d3, methylphenidate-d9, tramadol-d3, oxycodone-d3, and ephedrine-d3. All deuterated standards were from ReseaChem (Burgdorf, Switzerland), the solvents for washing and extraction were of analysis grade and obtained from Merck (Darmstadt, Germany); LC-solvents were of HPLC grade and were obtained from Sigma Aldrich (Buchs, Switzerland).

The LC-MS/MS apparatus was an ABSciex QTrap 3200 (Analyst software Version 1.5, Turbo V ion source operated in the ESI mode, gas 1, nitrogen (50psi); gas 2, nitrogen (60psi); ion spray voltage, 3500V; ion source temperature, 450°C; curtain gas, nitrogen (20psi) collision gas, medium), with a Shimadzu Prominence LC-system (Shimadzu CBM 20 A controller, two Shimadzu LC 20 AD pumps including a degasser, a Shimadzu SIL 20 AC autosampler and a Shimadzu CTO 20 AC column oven, Shimadzu, Duisburg, Germany). Gradient elution was performed on a separation column (Synergi 4µ POLAR-RP 80A, 150x2.0 with a POLAR-RP 4x2.0 Security Guard Cartridge, (Phenomenex, Aschaffenburg, Germany). The mobile phase consisted of 1mM ammonium formate buffer adjusted to pH 3,5 with formic acid (eluent A) and acetonitrile containing 1mM ammonium formate and 1mM formic acid (eluent B). The analysis was performed in MRM mode with two transitions per analyte and one transition for each deuterated internal standard, respectively.

***Supplementary Methods 3.*** Procedure

Due to technical problems 3 participants had no values for ADHD, IQ, and BDI (one of each group: control, *Decreaser,* and *Sustained User*). For those participants, missing values were replaced with their group mean.

Cognitive data for sustained attention (n=51) and working memory (n=63) performance was available for a subset of the included participants. For the RVP task, 28 controls, 12 *Decreasers*, and 11 *Sustained Users* had available data at both measurement points. The LNST, a test commonly used to test the verbal working memory, was completed by a subset of 63 participants (37 controls, 14 *Decreasers*, and 12 *Sustained Users*).

***Supplementary Methods 4.*** MRI data preprocessing and analysis

Main steps during MRI data preprocessing in FreeSurfer include: motion correction, skull-stripping (Segonne et al., 2004), automated Talairach transformation, with subsequent segmentation of the white matter (Fischl et al., 2002; Fischl et al., 2004), correction of intensity variations due to magnetic field inhomogeneities and placement of gray/white and gray/cerebrospinal fluid borders based on intensity gradients (Dale et al., 1999; Dale and Sereno, 1993). Within the longitudinal stream, instead of registration to Talairach space, an unbiased within-subject template space and image is then created using robust, inverse consistent registration (Reuter et al., 2010; 2012). After preprocessing all images were visually checked and cortical thickness and cortical surface area (CSA) was extracted at each vertex of the surface. To gain a more accurate estimation of CSA of the cortex the area measured at the gray/white and the gray/cerebrospinal fluid borders were averaged in this study.

For subcortical analysis, T1-weighted images for both time points were first pre-processed using the minc-bpipe-library pipeline. Major steps within this pipeline are: iterative N4 bias field correction (Tustison et al., 2010), image registration (Avants et al., 2008; Vincent et al., 2016), masking and brain extraction (Eskildsen et al., 2012), and field-of-view cropping in order to provide outputs in native space with their coordinate system reoriented in accordance with MNI space (re-oriented not re-gridded). These images were then further analyzed using the MAGeTbrain toolbox (Chakravarty et al., 2013). With the help of the MAGeTbrain toolbox we aimed to extract volumetric information from thalamus, globus pallidus, and the striatum. The MAGeTbrain segmentation algorithm employs a single atlas for these subcortical structures derived from histological data (Chakravarty et al., 2006). The labels from this atlas were then first propagated to a subset of the entire sample. This subset consisted of 21 participants (templates), an optimal number based on prior work (Pipitone et al., 2014). The purpose of these templates is to capture the variability within the data set in order to ameliorate the segmentation of each individual subject. Thus, the templates consisted of 10 controls and 11 CU with equal male to female ratio and age as the entire sample. Each template brain is then used to segment each subject leading to 21 labels which are then integrated by majority-label fusion (Collins and Pruessner, 2010). Each image was visually checked to ensure the accuracy of the segmentation.

In our study we refrained to weight the ROIs for the CSA when analyzing CT as we were afraid to blur information from CSA into our CT analysis. Moreover, from a statistical point of view, such a weighting would be only adequate if there is a real dependency between CT and CSA, e.g., when larger areas would show thicker or thinner cortices. However, usually there is no such relationship between CT and CSA and therefore it is also not recommended to use intracranial volume (ICV), total gray matter volume (TGMV), or total cortical surface area (TCSA) as a covariate of no interest when analyzing cortical thickness.

Moreover, as we apply the same procedure for all the groups we do not assume that this would have influenced our conclusion from the longitudinal results.

***Supplementary Methods 5.*** ROI-correlations between the right and the left hemisphere

*Cortical Thickness:*
Baseline: Superior frontal gyrus r=0.84; middle frontal gyrus r=0.76; inferior frontal gyrus r=0.54; lateral orbitofrontal gyrus r=0.60; medial orbitofrontal gyrus r=0.41; anterior cingulate cortex r=0.41; all P<0.001.
Follow-up: Superior frontal gyrus r=0.84; middle frontal gyrus r=0.79; inferior frontal gyrus r=0.79; medial orbitofrontal gyrus r=0.49; lateral orbitofrontal gyrus r=0.60; anterior cingulate cortex r=0.57; all P<0.001.

*Cortical Surface Area:*Baseline: Superior frontal gyrus r=0.87; middle frontal gyrus r=0.87; inferior frontal gyrus r=0.70; lateral orbitofrontal gyrus r=0.82; medial orbitofrontal gyrus r=0.58; anterior cingulate cortex r=0.42; all P<0.001.
Follow-up: Superior frontal gyrus r=0.87; middle frontal gyrus r=0.88; inferior frontal gyrus r=0.70; lateral orbitofrontal gyrus r=0.82; medial orbitofrontal gyrus r=0.57; anterior cingulate cortex r=0.39; all P<0.001.

*Subcortical Volumes:*Baseline: Thalamus r=0.96; Globus Pallidus r=0.93; Striatum r=0.98; all P<0.001.
Follow-up: Thalamus r=0.96; Globus Pallidus r=0.93; Striatum r=0.98; all P<0.001.

***Supplementary Table 1:*** Neuroanatomical group differences at baseline and follow-up

|  | | *Baseline* | | | | | |  |  | *Follow-up* | | | | | |
| --- | --- | --- | --- | --- | --- | --- | --- | --- | --- | --- | --- | --- | --- | --- | --- |
|  | | Controls (*n*=38) | Cocaine Users (*n*=29) | *F* | *df, df_err_* | *P* | *d* |  |  | Controls (*n*=38) | Cocaine Users (*n*=29) | *F* | *df, df_err_* | *P* | *d* |
|  | |  |  |  |  |  |  |  |  |  |  |  |  |  |  |
| **Cortical Thickness** | |  |  |  |  |  |  |  |  |  |  |  |  |  |  |
| Superior frontal gyrus | | 2.84 (0.02) | 2.75 (0.02) | 6.83 | 1, 62 | **0.011*** | 0.66 |  |  | 2.84 (0.02) | 2.77 (0.02) | 5.28 | 1, 62 | **0.025** | 0.58 |
| Middle frontal gyrus | | 2.50 (0.02) | 2.44 (0.02) | 3.81 | 1, 62 | 0.056 | 0.52 |  |  | 2.51 (0.02) | 2.45 (0.02) | 4.34 | 1, 62 | **0.041** | 0.54 |
| Inferior frontal gyrus | | 2.65 (0.02) | 2.58 (0.02) | 5.59 | 1, 62 | **0.021*** | 0.61 |  |  | 2.66 (0.02) | 2.59 (0.03) | 3.41 | 1, 62 | 0.069 | 0.49 |
| Medial OFC | | 2.39 (0.02) | 2.32 (0.03) | 2.67 | 1, 62 | 0.107 | 0.50 |  |  | 2.38 (0.02) | 2.34 (0.03) | 1.13 | 1, 62 | 0.293 | 0.30 |
| Lateral OFC | | 2.63 (0.02) | 2.54 (0.03) | 5.74 | 1, 62 | **0.02*** | 0.71 |  |  | 2.61 (0.02) | 2.53 (0.02) | 7.16 | 1, 62 | **0.01** | 0.72 |
| ACC | | 2.81 (0.02) | 2.78 (0.03) | 0.92 | 1, 62 | 0.341 | 0.27 |  |  | 2.80 (0.03) | 2.78 (0.03) | 0.30 | 1, 62 | 0.584 | 0.16 |
|  | |  |  |  |  |  |  |  |  |  |  |  |  |  |  |
| **Surface Area^a^** | |  |  |  |  |  |  |  |  |  |  |  |  |  |  |
| Superior frontal gyrus | | 8117.9 (69.1) | 8271.7 (81.5) | 1.71 | 1, 61 | 0.196 | 0.16 |  |  | 8069.6 (67.4) | 8214.8 (79.5) | 1.59 | 1, 61 | 0.212 | 0.16 |
| Middle frontal gyrus | | 9352.4 (94.7) | 9475.3 (111.6) | 0.58 | 1, 61 | 0.449 | 0.11 |  |  | 9273.2 (93.3) | 9381.4 (110.0) | 0.46 | 1, 61 | 0.499 | 0.10 |
| Inferior frontal gyrus | | 4166.8 (50.2) | 4301.6 (59.2) | 2.48 | 1, 61 | 0.121 | 0.29 |  |  | 4142.0 (49.7) | 4271.2 (58.7) | 2.32 | 1, 61 | 0.133 | 0.28 |
| Medial OFC | | 2155.5 (22.7) | 2160.7 (26.8) | 0.02 | 1, 61 | 0.895 | 0.02 |  |  | 2139.1 (22.5) | 2135.1 (26.6) | 0.01 | 1, 61 | 0.917 | 0.02 |
| Lateral OFC | | 2987.3 (32.4) | 2960.7 (38.2) | 0.23 | 1, 61 | 0.632 | 0.08 |  |  | 2957.8 (33.8) | 2949.9 (39.8) | 0.02 | 1, 61 | 0.891 | 0.02 |
| ACC | | 1802.1 (25.3) | 1649.0 (29.9) | 12.58 | 1, 61 | **0.001*** | 0.66 |  |  | 1789.5 (24.6) | 1642.9 (29.0) | 12.19 | 1, 61 | **0.001*** | 0.63 |
|  | |  |  |  |  |  |  |  |  |  |  |  |  |  |  |
| **Subcortical^b^** | |  |  |  |  |  |  |  |  |  |  |  |  |  |  |
| Thalamus | | 6628.3 (67.0) | 6384.9 (79.0) | 4.55 | 1, 61 | **0.037** | 0.46 |  |  | 6585.5 (74.8) | 6333.7 (88.1) | 3.91 | 1, 61 | 0.052 | 0.46 |
| GP | | 1465.3 (16.9) | 1416.6 (19.9) | 2.85 | 1, 61 | 0.096 | 0.40 |  |  | 1464.8 (18.6) | 1407.9 (21.9) | 3.23 | 1, 61 | 0.077 | 0.45 |
| Striatum | | 9935.7 (130.5) | 9648.6 (153.8) | 1.67 | 1, 61 | 0.201 | 0.33 |  |  | 9864.2 (134.7) | 9537.1 (158.8) | 2.03 | 1, 61 | 0.159 | 0.37 |
|  | |  |  |  |  |  |  |  |  |  |  |  |  |  |  |
|  | Estimated means (in mm (cortical thickness) /mm^2^ (cortical surface area) /mm^3^ (volume)) and standard errors.  All group comparisons were adjusted for age, IQ, and ADHD-SR score ^a^ additionally corrected for total surface area  ^b^ additionally corrected for ICV  Significant P values are shown in bold, * significant after FDR correction (Benjamini and Hochberg, 1995)  Abbreviations: ACC=anterior cingulate cortex; GP=globus pallidus; OFC=orbitofrontal cortex | | | | | | | | | | | | | | |

***Supplementary Table 2:*** Neuroanatomical group differences at baseline and follow-up with gender as an additional covariate

|  | | *Baseline* | | | | | |  |  | *Follow-up* | | | | | |
| --- | --- | --- | --- | --- | --- | --- | --- | --- | --- | --- | --- | --- | --- | --- | --- |
|  | | Controls (*n*=38) | Cocaine Users (*n*=29) | *F* | *df, df_err_* | *P* | *d* |  |  | Controls (*n*=38) | Cocaine Users (*n*=29) | *F* | *df, df_err_* | *P* | *d* |
|  | |  |  |  |  |  |  |  |  |  |  |  |  |  |  |
| **Cortical Thickness** | |  |  |  |  |  |  |  |  |  |  |  |  |  |  |
| Superior frontal gyrus | | 2.84 (0.02) | 2.75 (0.02) | 6.66 | 1, 61 | **0.012*** | 0.65 |  |  | 2.84 (0.02) | 2.77 (0.02) | 5.13 | 1, 61 | **0.027** | 0.56 |
| Middle frontal gyrus | | 2.5 (0.02) | 2.44 (0.02) | 3.70 | 1, 61 | 0.059 | 0.51 |  |  | 2.51 (0.02) | 2.45 (0.02) | 4.20 | 1, 61 | **0.045** | 0.53 |
| Inferior frontal gyrus | | 2.65 (0.02) | 2.58 (0.02) | 5.47 | 1, 61 | **0.023*** | 0.60 |  |  | 2.66 (0.02) | 2.59 (0.03) | 3.30 | 1, 61 | 0.074 | 0.47 |
| Medial OFC | | 2.39 (0.02) | 2.32 (0.03) | 2.65 | 1, 61 | 0.109 | 0.51 |  |  | 2.38 (0.02) | 2.34 (0.02) | 1.05 | 1, 61 | 0.310 | 0.28 |
| Lateral OFC | | 2.63 (0.02) | 2.54 (0.03) | 5.80 | 1, 61 | **0.019*** | 0.71 |  |  | 2.61 (0.02) | 2.53 (0.02) | 7.07 | 1, 61 | **0.010** | 0.71 |
| ACC | | 2.82 (0.02) | 2.77 (0.03) | 1.00 | 1, 61 | 0.321 | 0.29 |  |  | 2.8 (0.03) | 2.78 (0.03) | 0.31 | 1, 61 | 0.577 | 0.16 |
|  | |  |  |  |  |  |  |  |  |  |  |  |  |  |  |
| **Surface Area^a^** | |  |  |  |  |  |  |  |  |  |  |  |  |  |  |
| Superior frontal gyrus | | 8115.4 (68.6) | 8275.1 (80.9) | 1.87 | 1, 60 | 0.177 | 0.17 |  |  | 8066.4 (67.1) | 8219.1 (79.2) | 1.78 | 1, 60 | 0.187 | 0.16 |
| Middle frontal gyrus | | 9350.4 (95.0) | 9477.9 (112.0) | 0.62 | 1, 60 | 0.434 | 0.12 |  |  | 9270.5 (93.7) | 9384.9 (110.5) | 0.51 | 1, 60 | 0.477 | 0.10 |
| Inferior frontal gyrus | | 4168.5 (50.0) | 4299.4 (59.0) | 2.36 | 1, 60 | 0.130 | 0.28 |  |  | 4144.2 (49.6) | 4268.2 (58.5) | 2.15 | 1, 60 | 0.148 | 0.27 |
| Medial OFC | | 2155.3 (22.9) | 2161 (27.0) | 0.02 | 1, 60 | 0.884 | 0.03 |  |  | 2139.1 (22.7) | 2135.2 (26.8) | 0.01 | 1, 60 | 0.920 | 0.02 |
| Lateral OFC | | 2986.2 (32.3) | 2962.1 (38.1) | 0.19 | 1, 60 | 0.662 | 0.07 |  |  | 2956.1 (33.6) | 2952.1 (39.6) | 0.01 | 1, 60 | 0.944 | 0.01 |
| ACC | | 1801.3 (25.3) | 1650 (29.8) | 12.34 | 1, 60 | **0.001*** | 0.66 |  |  | 1788.4 (24.6) | 1644.3 (29.0) | 11.81 | 1, 60 | **0.001*** | 0.62 |
|  | |  |  |  |  |  |  |  |  |  |  |  |  |  |  |
| **Subcortical^b^** | |  |  |  |  |  |  |  |  |  |  |  |  |  |  |
| Thalamus | | 6629.0 (67.5) | 6384. (79.6) | 4.54 | 1, 60 | **0.037** | 0.46 |  |  | 6587.2 (75.0) | 6331.5 (88.4) | 4.01 | 1, 60 | 0.050 | 0.46 |
| GP | | 1466.0 (16.8) | 1415.8 (19.8) | 3.09 | 1, 60 | 0.084 | 0.41 |  |  | 1465.6 (18.4) | 1406.9 (21.6) | 3.52 | 1, 60 | 0.066 | 0.46 |
| Striatum | | 9939.0 (130.7) | 9644.3 (154.1) | 1.75 | 1, 60 | 0.191 | 0.34 |  |  | 9869.1 (133.9) | 9530.7 (157.9) | 2.2 | 1, 60 | 0.143 | 0.38 |
|  | |  |  |  |  |  |  |  |  |  |  |  |  |  |  |
|  | Estimated means (in mm (cortical thickness) /mm^2^ (cortical surface area) /mm^3^ (volume)) and standard errors.  All group comparisons were adjusted for age, IQ, ADHD-SR score, and gender ^a^ additionally corrected for total surface area  ^b^ additionally corrected for ICV  Significant P values are shown in bold, * significant after FDR correction (Benjamini and Hochberg, 1995)  Abbreviations: ACC= anterior cingulate cortex; GP=globus pallidus; OFC= orbitofrontal cortex | | | | | | | | | | | | | | |

| ***Supplementary Table 3:*** Neuroanatomical group differences at baseline between Decreasers and Sustained Users | | | | | | | | |
| --- | --- | --- | --- | --- | --- | --- | --- | --- |
|  | Decreasers (*n*=15) | Sustained Users (*n*=14) | *F* | *df, df_err_* | *P* | *d* |  |  |
|  |  |  |  |  |  |  |  |  |
| **Cortical Thickness** |  |  |  |  |  |  |  |  |
| Superior frontal gyrus | 2.77 (0.03) | 2.74 (0.03) | 0.46 | 1, 24 | 0.505 | 0.25 |  |  |
| Middle frontal gyrus | 2.44 (0.03) | 2.42 (0.03) | 0.18 | 1, 24 | 0.679 | 0.17 |  |  |
| Inferior frontal gyrus | 2.59 (0.03) | 2.58 (0.03) | 0.05 | 1, 24 | 0.829 | 0.09 |  |  |
| Medial OFC | 2.30 (0.03) | 2.38 (0.04) | 2.41 | 1, 24 | 0.134 | 0.6 |  |  |
| Lateral OFC | 2.54 (0.03) | 2.57 (0.03) | 0.21 | 1, 24 | 0.650 | 0.18 |  |  |
| ACC | 2.76 (0.04) | 2.81 (0.04) | 0.99 | 1, 24 | 0.329 | 0.39 |  |  |
|  |  |  |  |  |  |  |  |  |
| **Surface Area^a^** |  |  |  |  |  |  |  |  |
| Superior frontal gyrus | 7978.9 (109.2) | 8210 (113.4) | 1.94 | 1, 23 | 0.177 | 0.24 |  |  |
| Middle frontal gyrus | 9126.6 (130.1) | 9275.2 (135.2) | 0.56 | 1, 23 | 0.460 | 0.14 |  |  |
| Inferior frontal gyrus | 4125.5 (84.9) | 4332.2 (88.3) | 2.56 | 1, 23 | 0.123 | 0.48 |  |  |
| Medial OFC | 2153.2 (31.9) | 2103.5 (33.2) | 1.05 | 1, 23 | 0.317 | 0.23 |  |  |
| Lateral OFC | 2878.1 (42.8) | 2947.3 (44.5) | 1.13 | 1, 23 | 0.299 | 0.21 |  |  |
| ACC | 1656.2 (39.7) | 1635.9 (41.3) | 0.11 | 1, 23 | 0.739 | 0.09 |  |  |
|  |  |  |  |  |  |  |  |  |
| **Subcortical^b^** |  |  |  |  |  |  |  |  |
| Thalamus | 6447.1 (112.3) | 6297.0 (116.7) | 0.77 | 1, 23 | 0.388 | 0.27 |  |  |
| GP | 1453.0 (24.5) | 1411.8 (25.5) | 1.23 | 1, 23 | 0.280 | 0.33 |  |  |
| Striatum | 9908.5 (199.4) | 9456.9 (207.2) | 2.23 | 1, 23 | 0.149 | 0.49 |  |  |
|  |  |  |  |  |  |  |  |  |
| Estimated means (in mm (cortical thickness) /mm^2^ (cortical surface area) /mm^3^ (volume)) and standard errors.  All group comparisons were adjusted for age, IQ, and ADHD-SR score ^a^ additionally corrected for total surface area  ^b^ additionally corrected for ICV  Abbreviations: ACC=anterior cingulate cortex; GP=globus pallidus; OFC=orbitofrontal cortex | | | | | | | | |

| ***Supplementary Table 4:*** Associations between neuroanatomical structures and cocaine consumption parameters at baseline | | | | | | |
| --- | --- | --- | --- | --- | --- | --- |
|  | Region |  | Lifetime (g)^a^  (n=29) | Duration^a^  (n=29) | Grams/Week  (n=29) | Age of Onset  (n=29) |
|  |  |  |  |  |  |  |
| Thickness | Frontal^c^ | r | **-0.328** | **-0.346** | -0.001 | 0.140 |
|  |  | p | **0.04** | **0.04** | 0.50 | 0.23 |
| Surface Area^b^ | ACC | r | -0.048 | 0.020 | 0.088 | -0.102 |
|  |  | p | 0.41 | 0.46 | 0.33 | 0.30 |
|  | | | | | | |
| Pearson’s product-moment correlation coefficients, one-tailed  ^a^ adjusted for age  ^b^ additionally adjusted for total surface area  ^c^ mean of 3 ROIs which showed a significant group difference at baseline (superior, inferior frontal gyrus, and lateral orbitofrontal cortex)  Abbreviations: ACC=anterior cingulate cortex; g=gram  Significant results are shown in bold | | | | | | |

***Supplementary Table 5:*** Correlations between neuroanatomical structures and substance consumption at baseline

|  | Region |  | Alcohol (g) per week  (n=28) | Cigerettes per day  (n=29) | Cannabis Lifetime (g)^a^  (n=26) | Amphetamine Lifetime (g)^a^  (n=23) | MDMA Lifetime (tablet)^a^  (n=20) | MPH Lifetime (tablet)^a^  (n=8) |
| --- | --- | --- | --- | --- | --- | --- | --- | --- |
|  |  |  |  |  |  |  |  |  |
| Thickness | Frontal^c^ | r | 0.136 | -0.308 | -0.141 | 0.095 | 0.165 | -0.567 |
|  |  | p | 0.25 | 0.05 | 0.25 | 0.34 | 0.25 | 0.09 |
| Surface Area^b^ | ACC | r | 0.024 | 0.022 | -0.301 | 0.157 | 0.161 | 0.156 |
|  |  | p | 0.45 | 0.50 | 0.08 | 0.25 | 0.26 | 0.38 |
|  | | | | | | | | |
| Pearson’s product-moment correlation coefficients, one-tailed  ^a^ adjusted for age  ^b^ additionally adjusted for total surface area  ^c^ mean of 3 ROIs which showed significant group difference at baseline (superior, inferior frontal gyrus, and lateral orbitofrontal cortex)  Abbreviations: ACC=anterior cingulate cortex; g=gram; MDMA=3,4methylenedioxymethamphetamine (“ecstasy”); MPH=Methylphenidate | | | | | | | | |

***Supplementary Table 6:*** Correlations between changes in neuroanatomical structures and substance consumption

|  |  |  | Cumulative dose during interval | | | | |
| --- | --- | --- | --- | --- | --- | --- | --- |
|  | Region |  | Alcohol  (g)  (n=29) | Cannabis (g)  (n=23) | Amphetamine (g)  (n=15) | MDMA (tablet)  (n=19) | MPH (tablet)  (n=5) |
|  |  |  |  |  |  |  |  |
| Thickness | Frontal^a^ | r | -0.234 | -0.010 | -0.092 | -0.312 | 0.747 |
|  |  | p | 0.11 | 0.48 | 0.37 | 0.10 | 0.07 |
| \|  \|  \|  \|  \|  \| \| --- \| --- \| --- \| --- \| --- \|   Pearson’s product-moment correlation coefficients, one-tailed. ^a^ mean of 3 ROIs which showed a significant group*time interaction (superior, middle, and inferior frontal gyrus)  Abbreviations: g=gram; MDMA= 3,4-methylenedioxymethamphetamine (“ecstasy”); MPH=Methylphenidate | | | | | | | |
|  | | | | | | | |

***Supplementary Figure 1:***

***
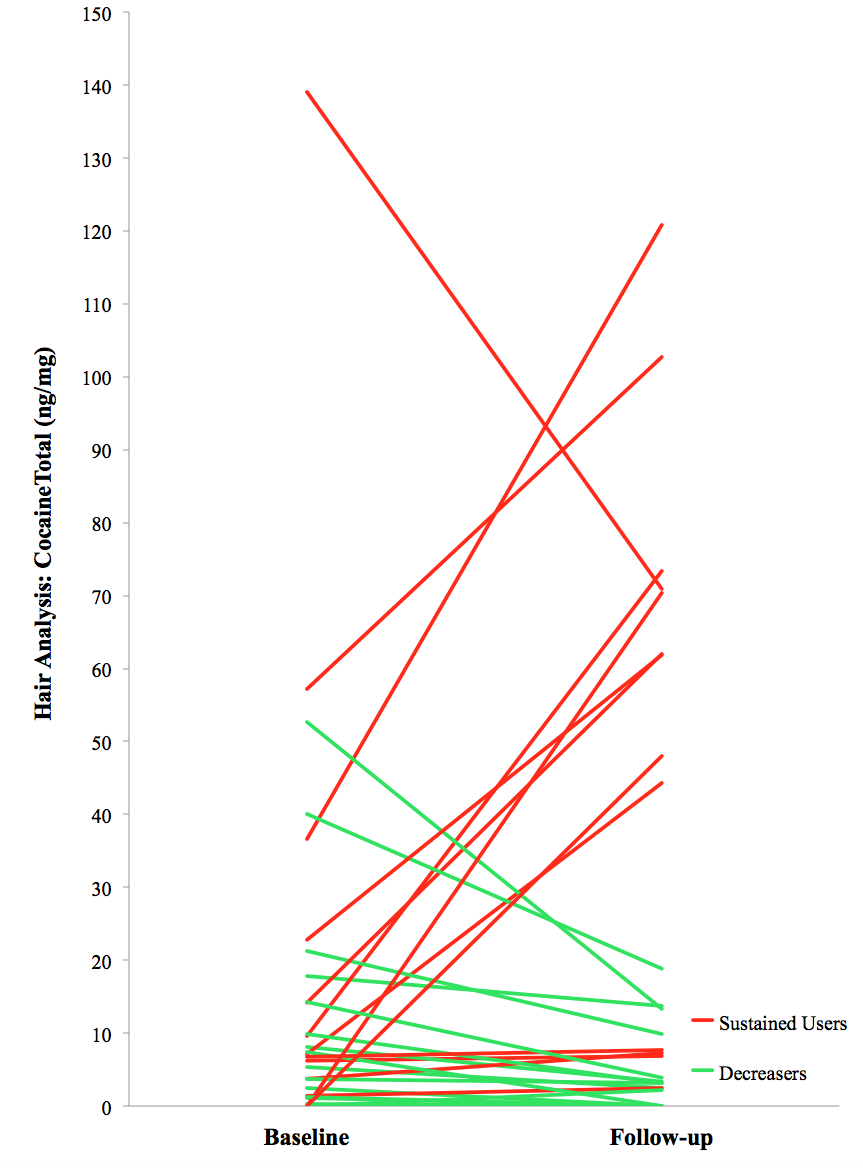
***Hair concentration levels of cocaine_total_ at baseline and follow-up in ng/mg.

***Supplementary Figure 2:***


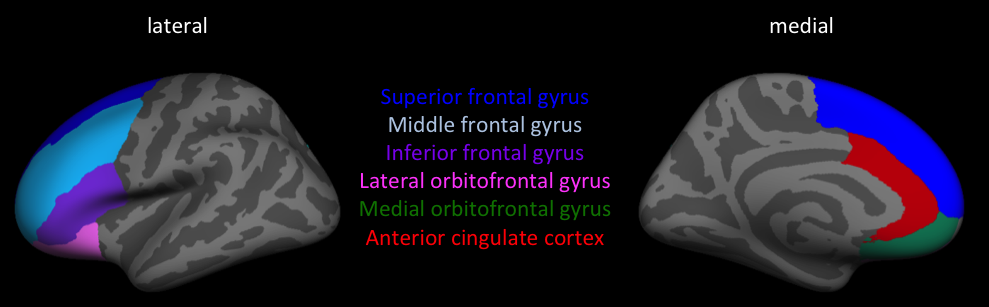
Supplementary Fig. 2 represents the inflated left hemisphere of the brain with the six regions of interest used. For the final analysis, cortical thickness and cortical surface values within these regions were averaged, summed up, respectively, across hemisphere.

***Supplementary Figure 3A:***


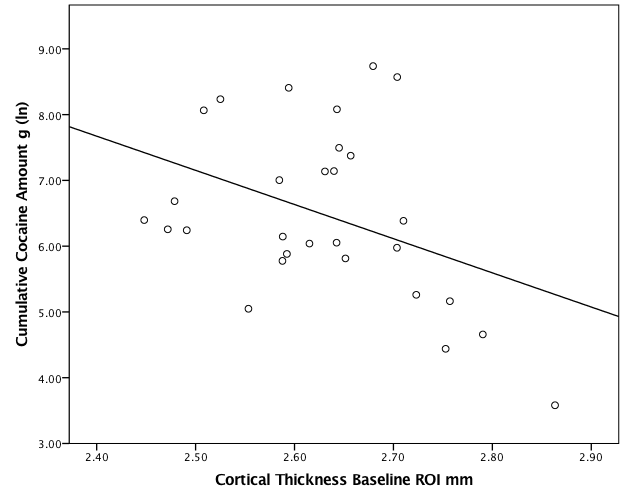


Partial correlations between ln-transformed lifetime cocaine use and cortical thickness measurement at baseline adjusted for age (n=29, r=-0.33, P=0.04; region of interest= average of cortical thickness within the superior frontal gyrus, inferior frontal gyrus and the lateral orbitofrontal gyrus).


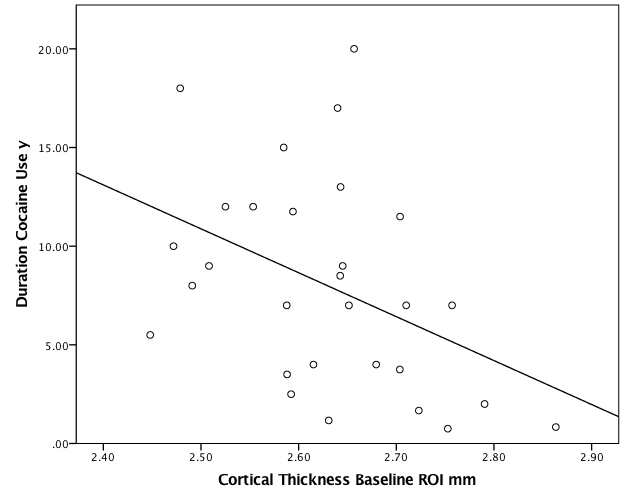
***Supplementary Figure 3B:***

Partial correlations between duration of cocaine use and cortical thickness measurement at baseline adjusted for age (n=29, r=-0.35, P=0.04; region of interest= average of cortical thickness within the superior frontal gyrus, inferior frontal gyrus and the lateral orbitofrontal gyrus).

***Supplementary Figure 4:***


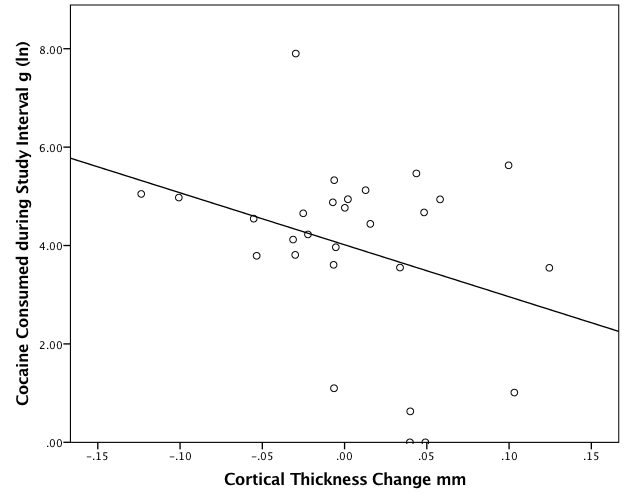


Correlation between the amount of cocaine consumed during the two measurement points and change in cortical thickness within the lateral frontal lobe (for both measures Δ_t2-t1_; n=28, r=-0.32, P=0.047; region of interest= average of cortical thickness within the superior, middle, and inferior frontal gyrus).

***Supplementary Figure 5:***


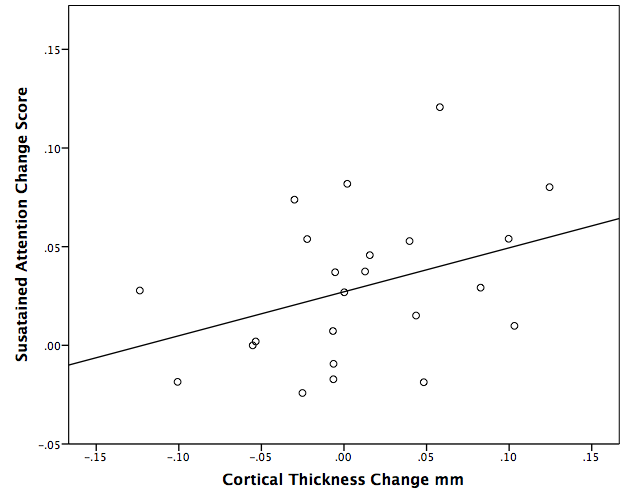


Correlation between sustained attention change score and change in cortical thickness within the lateral frontal lobe (for both measures Δ_t2-t1_; n=23, r=0.36, P=0.044; region of interest= average of cortical thickness within the superior, middle, and inferior frontal gyrus).

**Supplementary References:**

Avants, B.B., Epstein, C.L., Grossman, M., Gee, J.C., 2008. Symmetric diffeomorphic image registration with cross-correlation: evaluating automated labeling of elderly and neurodegenerative brain. Med Image Anal 12, 26-41.

Benjamini, Y., Hochberg, Y., 1995. Controlling the false discovery rate: a practical and powerful approach to multiple testing. . Journal of the Royal Statistical Society. 57, 289-300.

Chakravarty, M.M., Bertrand, G., Hodge, C.P., Sadikot, A.F., Collins, D.L., 2006. The creation of a brain atlas for image guided neurosurgery using serial histological data. Neuroimage 30, 359-376.

Chakravarty, M.M., Steadman, P., van Eede, M.C., Calcott, R.D., Gu, V., Shaw, P., Raznahan, A., Collins, D.L., Lerch, J.P., 2013. Performing label-fusion-based segmentation using multiple automatically generated templates. Hum Brain Mapp 34, 2635-2654.

Collins, D.L., Pruessner, J.C., 2010. Towards accurate, automatic segmentation of the hippocampus and amygdala from MRI by augmenting ANIMAL with a template library and label fusion. Neuroimage 52, 1355-1366.

Dale, A.M., Fischl, B., Sereno, M.I., 1999. Cortical surface-based analysis. I. Segmentation and surface reconstruction. Neuroimage 9, 179-194.

Dale, A.M., Sereno, M.I., 1993. Improved Localizadon of Cortical Activity by Combining EEG and MEG with MRI Cortical Surface Reconstruction: A Linear Approach. J Cogn Neurosci 5, 162-176.

Engeli, E.J.E., Zoelch, N., Hock, A., Nordt, C., Hulka, L.M., Kirschner, M., Esposito, F., Baumgartner, M.R., Henning, A., Seifritz, E., Quednow, B.B., Herdener, M., in preparation. Metabolic changes in nucleus accumbens in the craving brain: the role of glutamate in cocaine addiction.

Eskildsen, S.F., Coupe, P., Fonov, V., Manjon, J.V., Leung, K.K., Guizard, N., Wassef, S.N., Ostergaard, L.R., Collins, D.L., Alzheimer's Disease Neuroimaging, I., 2012. BEaST: brain extraction based on nonlocal segmentation technique. Neuroimage 59, 2362-2373.

Fischl, B., Salat, D.H., Busa, E., Albert, M., Dieterich, M., Haselgrove, C., van der Kouwe, A., Killiany, R., Kennedy, D., Klaveness, S., Montillo, A., Makris, N., Rosen, B., Dale, A.M., 2002. Whole brain segmentation: automated labeling of neuroanatomical structures in the human brain. Neuron 33, 341-355.

Fischl, B., Salat, D.H., van der Kouwe, A.J., Makris, N., Segonne, F., Quinn, B.T., Dale, A.M., 2004. Sequence-independent segmentation of magnetic resonance images. Neuroimage 23 Suppl 1, S69-84.

Kirschner, M., Sladky, R., Haugg, A., Stampfli, P., Jehli, E., Hodel, M., Engeli, E., Hosli, S., Baumgartner, M.R., Sulzer, J., Huys, Q.J.M., Seifritz, E., Quednow, B.B., Scharnowski, F., Herdener, M., 2018. Self-regulation of the dopaminergic reward circuit in cocaine users with mental imagery and neurofeedback. EBioMedicine 37, 489-498.

Pipitone, J., Park, M.T., Winterburn, J., Lett, T.A., Lerch, J.P., Pruessner, J.C., Lepage, M., Voineskos, A.N., Chakravarty, M.M., Alzheimer's Disease Neuroimaging, I., 2014. Multi-atlas segmentation of the whole hippocampus and subfields using multiple automatically generated templates. Neuroimage 101, 494-512.

Preller, K.H., Herdener, M., Schilbach, L., Stampfli, P., Hulka, L.M., Vonmoos, M., Ingold, N., Vogeley, K., Tobler, P.N., Seifritz, E., Quednow, B.B., 2014. Functional changes of the reward system underlie blunted response to social gaze in cocaine users. Proc Natl Acad Sci U S A 111, 2842-2847.

Reuter, M., Rosas, H.D., Fischl, B., 2010. Highly accurate inverse consistent registration: a robust approach. Neuroimage 53, 1181-1196.

Reuter, M., Schmansky, N.J., Rosas, H.D., Fischl, B., 2012. Within-subject template estimation for unbiased longitudinal image analysis. Neuroimage 61, 1402-1418.

Segonne, F., Dale, A.M., Busa, E., Glessner, M., Salat, D., Hahn, H.K., Fischl, B., 2004. A hybrid approach to the skull stripping problem in MRI. Neuroimage 22, 1060-1075.

Tustison, N.J., Avants, B.B., Cook, P.A., Zheng, Y., Egan, A., Yushkevich, P.A., Gee, J.C., 2010. N4ITK: improved N3 bias correction. IEEE Trans Med Imaging 29, 1310-1320.

Vincent, R.D., Neelin, P., Khalili-Mahani, N., Janke, A.L., Fonov, V.S., Robbins, S.M., Baghdadi, L., Lerch, J., Sled, J.G., Adalat, R., MacDonald, D., Zijdenbos, A.P., Collins, D.L., Evans, A.C., 2016. MINC 2.0: A Flexible Format for Multi-Modal Images. Front Neuroinform 10, 35.

Vonmoos, M., Hulka, L.M., Preller, K.H., Minder, F., Baumgartner, M.R., Quednow, B.B., 2014. Cognitive impairment in cocaine users is drug-induced but partially reversible: evidence from a longitudinal study. Neuropsychopharmacology 39, 2200-2210.
